# Mitochondrial LonP1 protease is implicated in the degradation of unstable Parkinson's disease-associated *DJ-1/PARK 7* missense mutants

**DOI:** 10.1038/s41598-021-86847-2

**Published:** 2021-04-01

**Authors:** Raúl Sánchez-Lanzas, José G. Castaño

**Affiliations:** 1 Departamento de Bioquímica, UAM-CSIC, Facultad de Medicina UAM, 28029 Madrid, Spain; 2 Instituto de Investigaciones Biomédicas “Alberto Sols”, UAM-CSIC, Facultad de Medicina UAM, 28029 Madrid, Spain

**Keywords:** Biochemistry, Cell biology, Molecular biology, Neuroscience, Pathogenesis

## Abstract

*DJ-1/PARK7* mutations are linked with familial forms of early-onset Parkinson's disease (PD). We have studied the degradation of untagged DJ-1 wild type (WT) and missense mutants in mouse embryonic fibroblasts obtained from DJ-1-null mice, an approach closer to the situation in patients carrying homozygous mutations. The results showed that the mutants L10P, M26I, A107P, P158Δ, L166P, E163K, and L172Q are unstable proteins, while A39S, E64D, R98Q, A104T, D149A, A171S, K175E, and A179T are as stable as DJ-1 WT. Inhibition of proteasomal and autophagic-lysosomal pathways had little effect on their degradation. Immunofluorescence and biochemical fractionation studies indicated that M26I, A107P, P158Δ, L166P, E163K, and L172Q mutants associate with mitochondria. Silencing of mitochondrial matrix protease LonP1 produced a strong reduction of the degradation of the mitochondrial-associated DJ-1 mutants A107P, P158Δ, L166P, E163K, and L172Q but not of mutant L10P. These results demonstrated a mitochondrial pathway of degradation of those DJ-1 missense mutants implicated in PD pathogenesis.

## Introduction

Most of the Parkinson’s disease (PD) patients are sporadic cases; rare familiar forms of PD can account for 5–10% of all PD cases. Mutations in several genes are well established as a genetic cause in familial PD^1^. *PARK7 /DJ-1* is one of those PD-linked genes whose pathogenic mutations show autosomal recessive inheritance and early-onset of the PD phenotype^2^. Those genetic mutations include CNVs**,** exonic deletions and truncations, splice-site mutations, homozygous (L10P, M26I, E64D, P158**Δ**, E163K, L166P and L172Q) and heterozygous (A39S, A104T and D149A) missense mutations, and rare polymorphisms (R98Q, A171S) in healthy individuals that are not associated with PD^1,3^.

Native DJ-1 protein is a dimer with a flavodoxin-like helix-strand-helix structure^4–9^. DJ-1, initially identified as a potential oncogene cooperating with Ha-Ras in cell transformation^10^, is implicated in several pathways, such as: transcriptional regulation^11–15^, RNA binding^16,17^, regulation of sumoylation^18^, protein folding as a chaperon^19–21^ or co-chaperon^22^ and cell death^23,24^. DJ-1 protein is cytoprotective being a sensor of oxidative stress and acting as antioxidant preventing apoptosis^25–35^.

Taking into account that some *PARK7/DJ-1* gene mutations result in a loss of function of the gene (no protein produced), it is reasonable to hypothesize that some of the missense point mutants may produce a loss of function of the protein. In agreement with this hypothesis, accelerated protein degradation (increased protein instability in the cell) of the DJ-1 point mutants would produce a decrease in the steady-state levels of the protein mimicking, in part, the phenotype of loss of gene function (no protein produced). In fact, DJ-1 L166P mutant has reduced stability in the cell^2,36–43^. DJ-1 M26I mutant have increased degradation in the cell according to some reports^40,41^, while other groups found no effect of the M26I mutation on protein stability^13,38,43,44^. The mutants A104T and D149A have also increased rates of degradation^41^, but found stable by other groups^13,38,43–45^. More recently described missense mutants of DJ-1 L10P, P158**Δ** and L172Q are also unstable proteins^46–49^. Finally, the point mutant E163K retains similar properties to wild type DJ-1 (WT) protein respect to stability in cells^50^, but it reduces the thermal stability of DJ-1 in solution disrupting a salt bridge of E163 with R145^51^.

Several studies of the degradation of DJ-1 missense mutants use tagged versions of the mutants and transfection into recipient cells expressing their endogenous DJ-1 WT. From our point of view the use of tagged constructs is inadequate to study protein degradation. When a protein is tagged either in its N-terminus or its C-terminus it is assumed that the behaviour of this protein in the cell will be equivalent to that of the untagged endogenous protein, but this assumption is not necessarily true. As a proof of the previous statement, N-terminal tagged DJ-1 L166P has a reduced degradation rate compared to untagged DJ-1 L166P, while tagging L166P at the C-terminus has less effect^52^. Another caveat to correctly interpret the results reported is that the DJ-1 missense mutants variably heterodimerize with the endogenous DJ-1 WT of the recipient cells, and those interactions may also, in principle, modify the stability of the mutant protein. This situation will not occur in patient cells where only the missense mutant DJ-1 protein is expressed (homozygous mutation). A way to circumvent those caveats is the study of the stability of the untagged DJ-1 missense mutants in DJ-1-null cells. The use of DJ-1-null mouse embryonic fibroblasts (MEFs) to study DJ-1 missense mutants stability have been used in some reports^47,48^. Here we have systematically investigated the degradation of untagged human wild type DJ-1 and its missense point variants: L10P^53^, M26I^54^, A39S^55^, E64D^56^, R98Q^54^, A104T^57^, A107P^58^, D149A^54^, P158**Δ**^58^, E163K^59^, A171S^57^, L172Q^49^, K175E^60^ and A179T^58,60^ in MEFs from DJ-1-null mice. The results have shown that DJ-1 pathogenic point mutants L10P, M26I, A107P, P158**Δ**, E163K, L166P and L172Q showed a significant increase of the degradation rate respect to DJ-1 WT in MEFs from DJ-1-null mice, and no significant difference respect to WT was found for A39S, E64D, A104T, D149A, K175E, A179T missense mutants or the rare R98Q, A171S polymorphic variants.

## Results

### Degradation of human wild type DJ-1 and missense mutants

**Figure 1:**
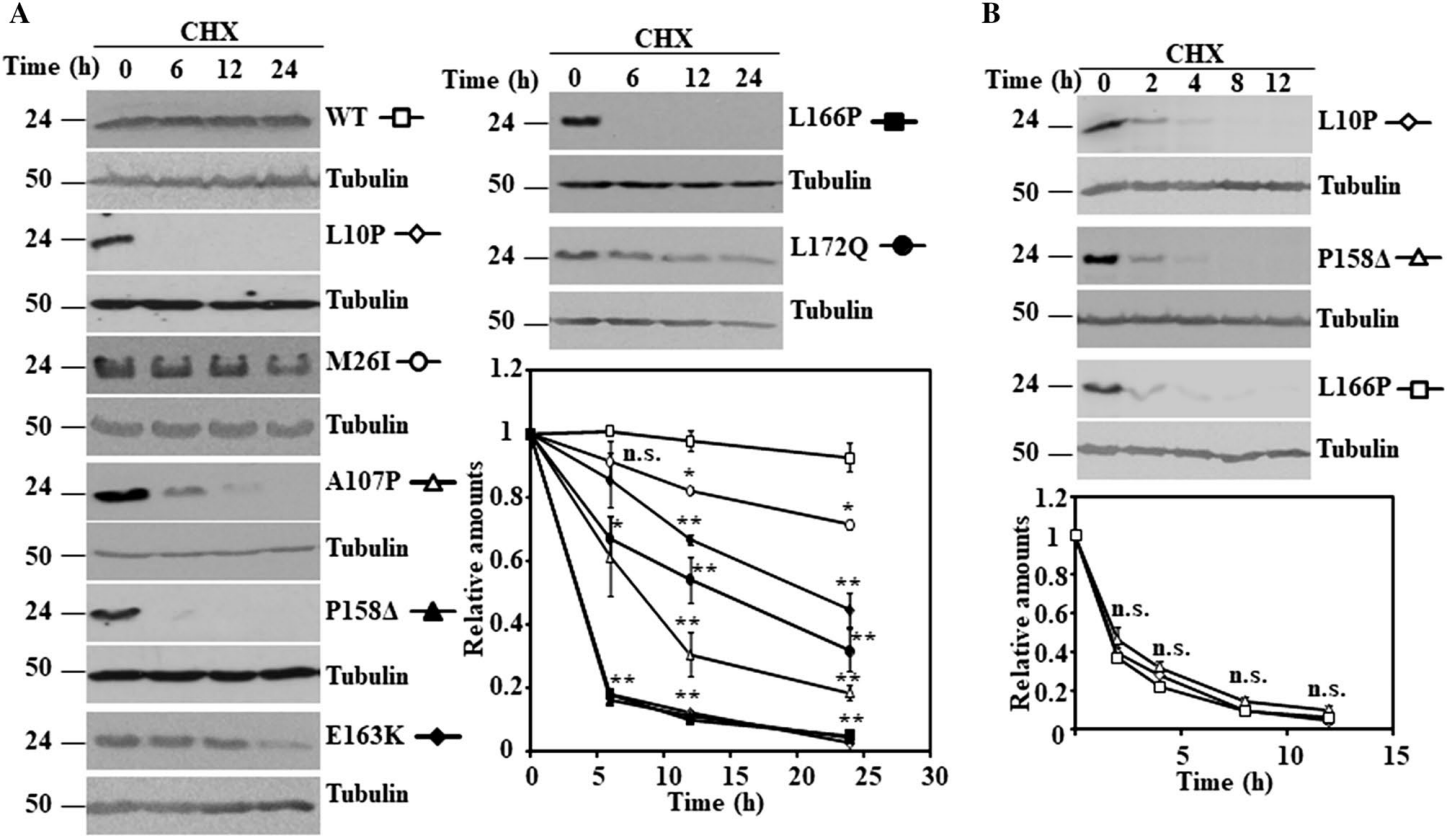
Time-course of the degradation of human DJ-1 unstable missense mutants transfected in DJ-1-null MEFs. DJ-1-null MEFs were transiently transfected with the indicated untagged human DJ-1 (hDJ-1) constructs and 48 h after transfection were treated with 25 µg/mL cycloheximide (CHX) for the times indicated. (**A**) Panels show representative immunoblots probed with anti-DJ-1 polyclonal antibodies of cells transfected with hDJ-1 WT and the missense mutants L10P, M26I, A107P, P158Δ, E163K, L166P and L172Q. (**B**) Panels show the results obtained with transfected DJ-1 missense mutants L10P, P158Δ and L166P in cells treated with CHX for shorter times, as indicated. Anti-tubulin antibodies were used as total protein loading control. Quantifications from three different experiments are shown in the graphs as mean ± s.e.m. Significant differences were found between hDJ-1 WT and hDJ-1 L10P at time points 6 h (**p = 8E−07), 12 h (**p = 1E−06) and 24 h (**p = 0.0008), between hDJ-1 WT and hDJ-1 M26I at time points 12 h (**p = 0.004) and 24 h (**p = 0.002), between hDJ-1 WT and hDJ-1 A107P at time points 6 h (*p = 0.03), 12 h (**p = 0.0007) and 24 h (**p = 2E−05), between hDJ-1 WT and hDJ-1 P158Δ at time points 6 h (**p = 4E−08), 12 h (**p = 1E−06) and 24 h (**p = 0.0008), between hDJ-1 WT and hDJ-1 E163K at time points 12 h (**p = 0.0001) and 24 h (**p = 0.001), between hDJ-1 WT and hDJ-1 L166P at time points 6 h (**p = 4E−08), 12 h (**p = 1E−06) and 24 h (**p = 5E−06) and between hDJ-1 WT and hDJ-1 L172Q at time points 6 h (*p = 0.04), 12 h (**p = 0.004) and 24 h (**p = 0.001). n.s., not significant.

**Figure 2:**
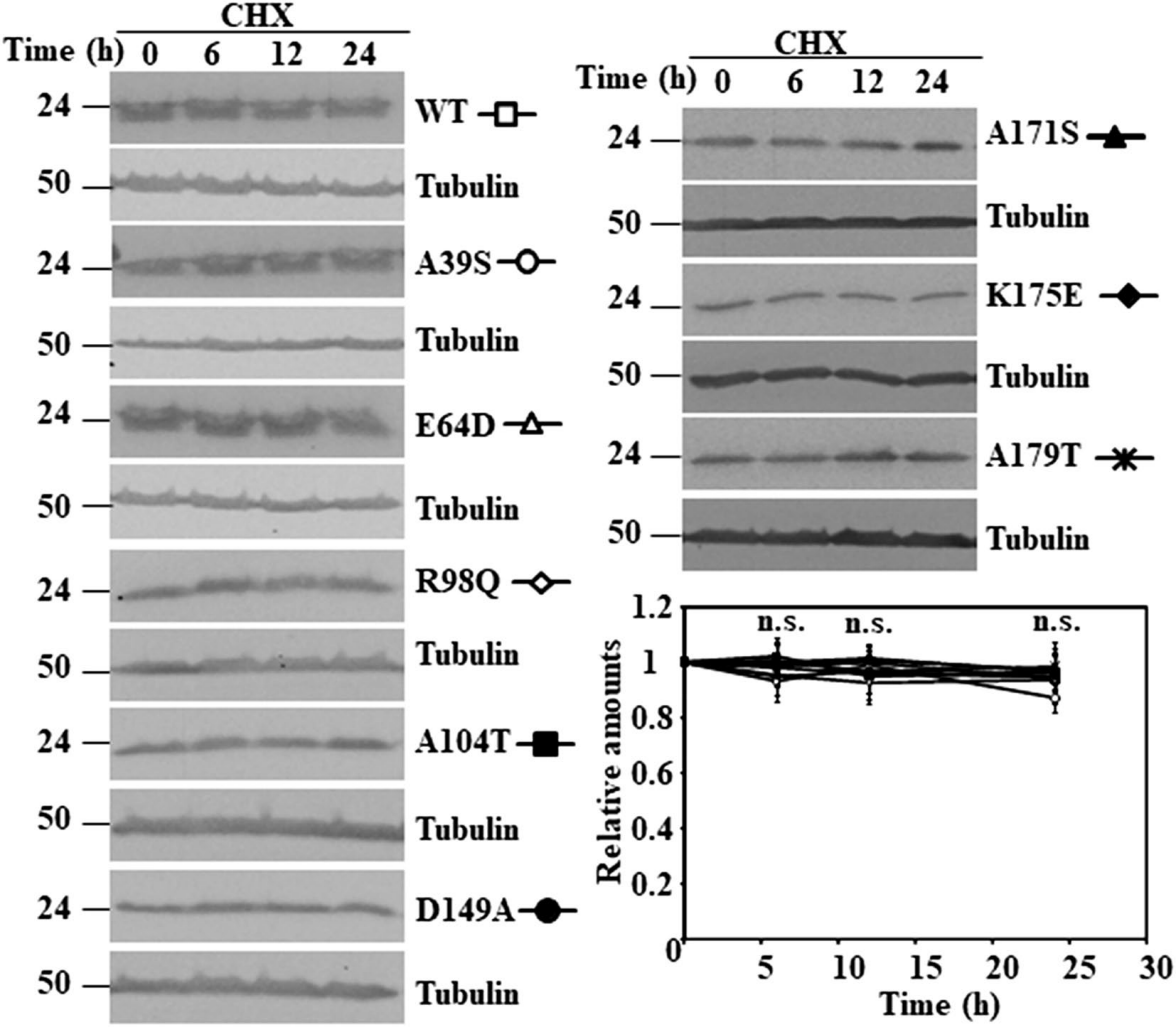
Degradation of human wild type DJ-1 and missense mutants in transfected DJ-1-null MEFs. DJ-1-null MEFs were transiently transfected with the indicated untagged human DJ-1 (hDJ-1) constructs and 48 h after transfection were treated with 25 µg/mL cycloheximide (CHX) for the times indicated. Panels show representative immunoblots developed with anti-DJ-1 polyclonal antibodies of human wild type DJ-1 (WT), A39S, E64D, R98Q, A104T, D149A, A171S, K175E and A179T. Anti-tubulin antibodies were used as total protein loading control. Below is shown the graph of quantification of the corresponding immunoblots. Data are mean ± s.e.m from three different experiments. *n.s.* not significant.

As previously stated in the introduction, degradation of ectopically expressed DJ-1 missense mutants could be affected by interactions with the cell endogenous DJ-1 and/or the use of tagged constructs. Accordingly, untagged wild type DJ-1 (WT) and the missense mutants were transfected into DJ-1-null mouse embryonic fibroblasts (MEFs). The mutants L10P, M26I, A107P, P158**Δ**, E163K, L166P and L172Q were unstable (Fig. 1A,B) after treatment of cells with cycloheximide (CHX) and showed different degradation rates. In contrast, DJ-1 WT and the missense mutants A39S, E64D, R98Q, A104T, D149A, A171S, K175E and A179T were not significantly degraded after treatment of the cells for 24 h with CHX (Fig. 2). The apparent degradation rate of DJ-1 missense mutants, evaluated by the corresponding half-life and ordered from shortest to longest half-life, was: L10P, P158**Δ** and L166P < A107P < L172Q < E163K < M26I (Supplementary Table I). We have previously shown that transfected human DJ-1 WT and point mutants M26I, R98Q, A104T, D149A are stable proteins in N2a mouse cells (expressing the endogenous mouse DJ-1) and L166P is unstable^43^. We extended those previous studies with similar assays to other missense DJ-1 mutants in the same cell line. In these experiments, the protein levels of the missense mutants A39S, A171S, K175E and A179T did not significantly change after treatment of transfected cells with CHX for 24 h (Supplementary Fig. 1A). In contrast, the point mutants L10P, A107P, P158**Δ**, E163K, L166P (shown again for comparison) and L172Q were degraded at different rates (Supplementary Fig. 1B).

**Figure 3:**
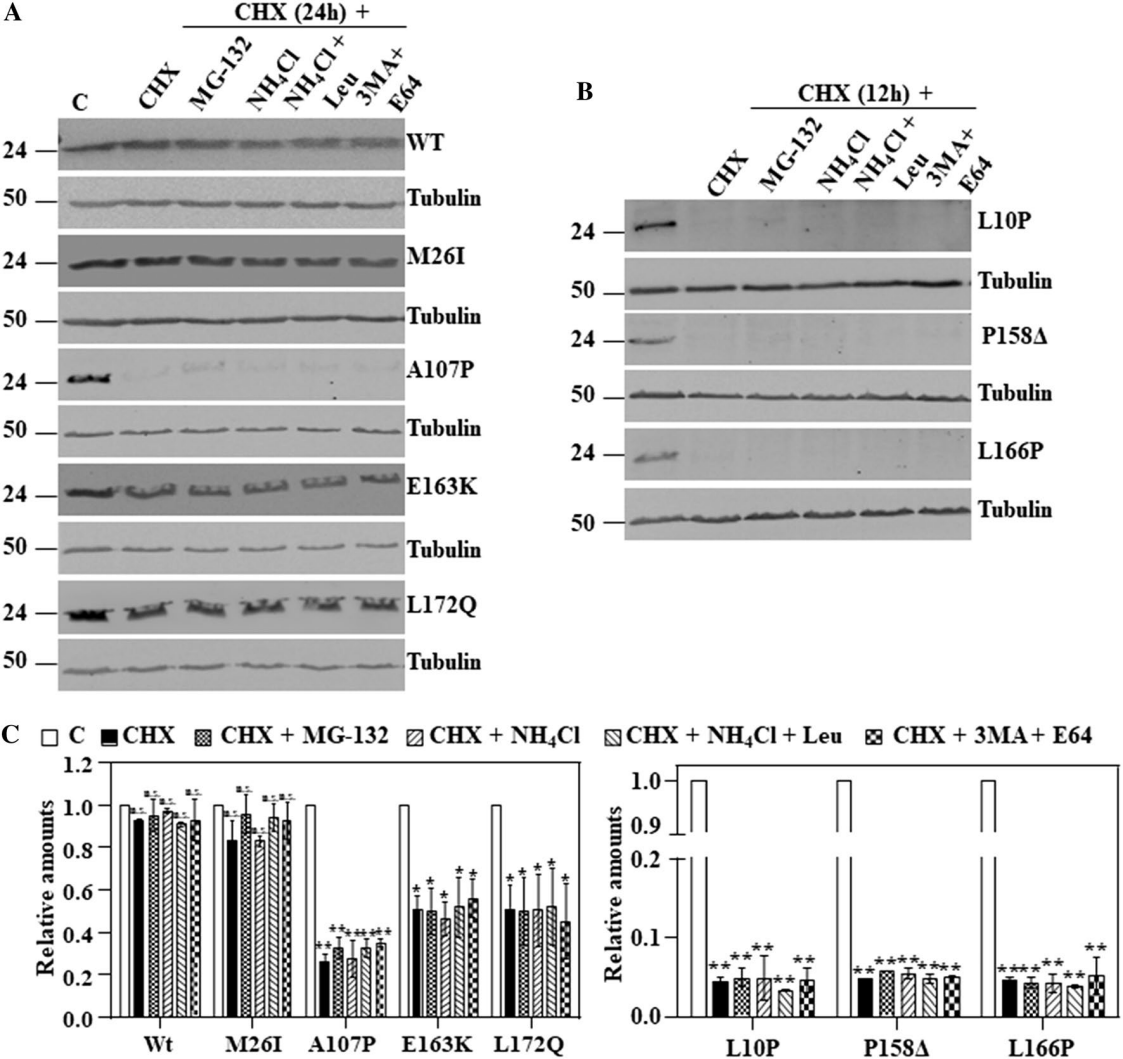
Effect of inhibitors of the proteasomal and autophagic-lysosomal pathways on the degradation of human wild type DJ-1 and missense mutants transfected in DJ-1-null MEFs. DJ-1-null MEFs were transiently transfected with the indicated untagged human DJ-1 (hDJ-1) constructs and 48 h after transfection were kept in complete medium (C) or treated with 25 µg/mL cycloheximide (CHX) in the absence (DMSO) or the presence of 10 µM MG-132, 20 mM NH_4_Cl, 20 mM NH_4_Cl plus 50 µM leupeptin (Leu) or 10 mM 3-methyl adenine (3MA) plus 5 µM E64 for 12 or 24 h, as indicated. Total cell lysates were analysed by Western and immunoblot with the corresponding specific antibodies. (**A**) Panels show representative immunoblots with anti-DJ-1 polyclonal antibody of human wild type DJ-1 (WT), M26I, A107P, E163K and L172Q. (**B**) Panels show the results obtained with transfected hDJ-1 L10P, P158Δ and L166P developed with anti-DJ-1 polyclonal antibody. Anti-tubulin antibodies were used as total protein loading control. (**C**) Graphs below each panel show the quantifications of the levels of DJ-1 protein respect to untreated cells as control. Values are expressed as mean ± s.e.m from three different experiments. Significant differences were found for hDJ-1 A107P between control cells and cells treated with CHX (**p = 0.002), CHX in combination with MG-132 (**p = 0.004), CHX in combination with NH_4_Cl (**p = 0.005), CHX in combination with NH_4_Cl plus leupeptin (**p = 0.003) and CHX in combination with 3MA plus E64 (**p = 0.001); for hDJ-1 E163K between control cells and cells treated with CHX (*p = 0.01), CHX in combination with MG-132 (*p = 0.03), CHX in combination with NH_4_Cl (*p = 0.02), CHX in combination with NH_4_Cl plus leupeptin (*p = 0.01) and CHX in combination with 3MA plus E64 (*p = 0.03); for hDJ-1 L172Q between control cells and cells treated with CHX (*p = 0.01), CHX in combination with MG-132 (*p = 0.03), CHX in combination with NH_4_Cl (*p = 0.04), CHX in combination with NH_4_Cl plus leupeptin (*p = 0.04) and CHX in combination with 3MA plus E64 (*p = 0.04); for hDJ-1 L10P between control cells and cells treated with CHX (**p = 0.0001), CHX in combination with MG-132 (**p = 6E−05), CHX in combination with NH_4_Cl (**p = 0.0003), CHX in combination with NH_4_Cl plus leupeptin (**p = 9E−05) and CHX in combination with 3MA plus E64 (**p = 0.0002); for hDJ-1 P158Δ between control cells and cells treated with CHX (**p = 5E−06), CHX in combination with MG-132 (**p = 9E−06), CHX in combination with NH_4_Cl (**p = 1E−05), CHX in combination with NH_4_Cl plus leupeptin (**p = 1E−05) and CHX in combination with 3MA plus E64 (**p = 4E−07) and for hDJ-1 L166P between control cells and cells treated with CHX (**p = 3E−06), CHX in combination with MG-132 (**p = 2E−05), CHX in combination with NH_4_Cl (**p = 5E−05), CHX in combination with NH_4_Cl plus leupeptin (**p = 2E−06) and CHX in combination with 3MA plus E64 (**p = 0.0002). *n.s.* not significant.

Although the proteasomal pathway is implicated in the degradation of DJ-L166P, its degradation is only partially prevented by proteasome inhibitors^43^. Therefore, we decided to re-evaluate those results and to test if inhibition of other proteolytic pathways can be more effective. To that end, transfected cells were treated with CHX for 24 h for those missense mutants that have shown longer half-lives: M26I, A107P, E163K and L172Q (Fig. 3A) and for 12 h for those missense mutants with shorter half-lives: L10P, P158**Δ** and L166P (Fig. 3B), in the absence or in the presence of several protease inhibitors. As shown in Fig. 3, addition of MG132 (proteasome inhibitor) or inhibitors of the autophagic-lyssosomal pathway (NH_4_Cl, NH_4_Cl in combination with leupeptin or 3-methyl adenine together with E64) had no significant effect on the degradation of the missense mutants or DJ-1 WT. Those results suggested that neither the proteasomal nor the autophagic-lysosomal pathways of protein degradation played a major role in the degradation of the unstable DJ-1 missense mutants.

### Subcellular localization of human wild type DJ-1 and missense mutants

**Figure 4:**
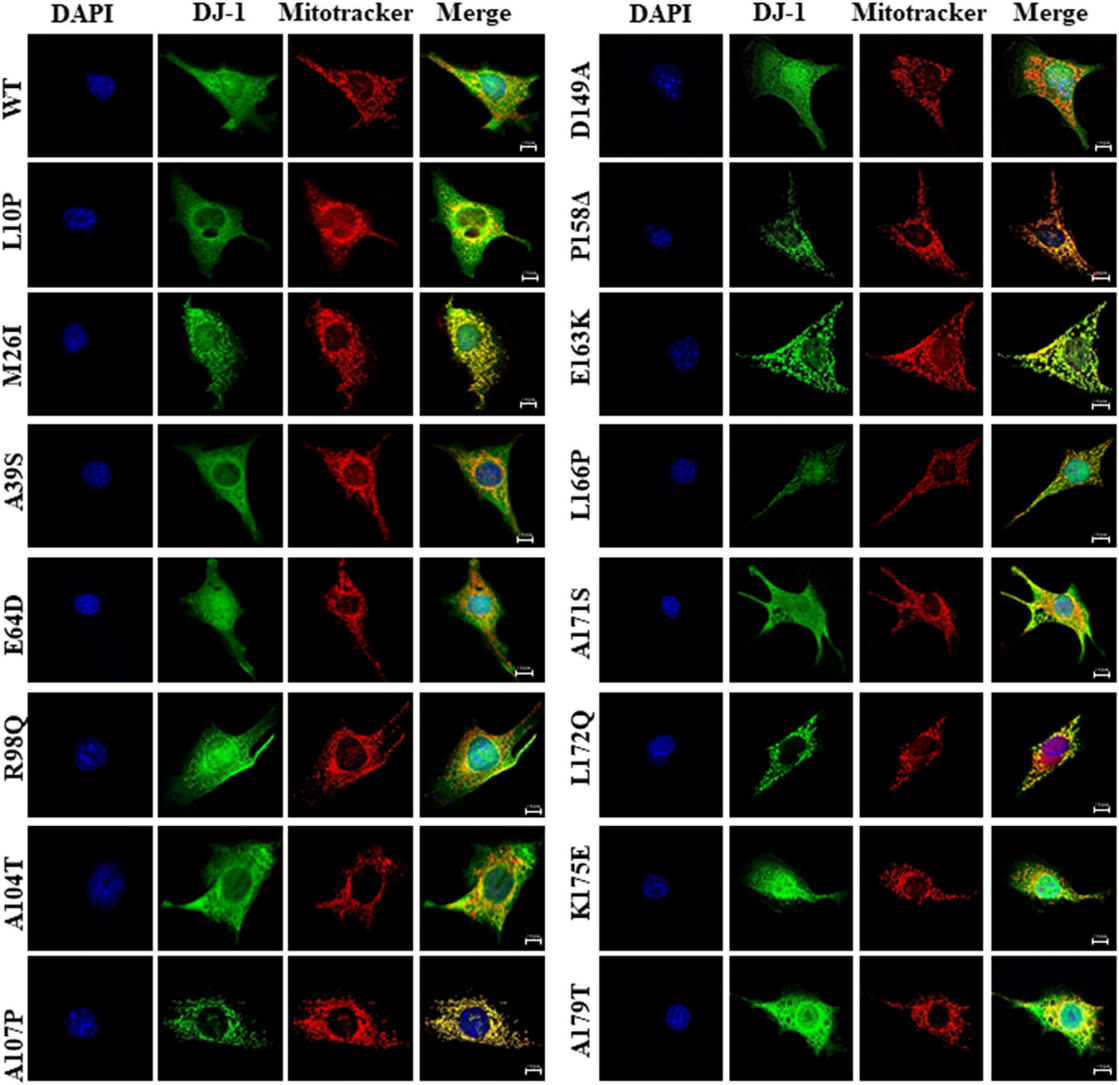
Subcellular localization by indirect immunofluorescence of human wild type DJ-1 and missense mutants transfected in DJ-1-null MEFs. Confocal fluorescence images of the indicated untagged human DJ-1 constructs in transfected DJ-1-null MEFs grown under basal conditions (complete medium), stained with Mitotracker (red), processed for immunofluorescence with anti-DJ-1 polyclonal specific antibodies (green) and counterstained with DAPI for nuclei visualization (blue). Quantifications of the co-localization of red (mitochondria) and green (DJ-1 immunofluorescence) pixels are presented in Supplementary Fig. 2.

A possible clue to understand the pathway of degradation of DJ-1 missense mutants could be their subcellular localization. DJ-1 protein is described to be localized both in the cytoplasm and in the nucleus^2,10,61^ and to translocate to the mitochondria upon cell oxidative stress^45,62^. L166P missense mutant is reported to be localized in the cytoplasm and mitochondria^2,63^, E163K mutant was shown to be localized in the mitochondria^63,64^, as well as M26I^63^ and L172Q^49^ missense mutants. Therefore, the subcellular localization of the human DJ-1 WT and missense mutants was determined after transfection of DJ-1-null MEFs by indirect immunofluorescence. Figure 4 shows the results obtained and Supplementary Fig. 2 shows the analysis of the co-localization of DJ-1 and mitotracker fluorescence. Without doubt, it can be concluded that the immunofluorescence signal of M26I, A107P, P158**Δ**, E163K, L166P and L172Q co-localize with mitotracker fluorescence (Pearson coefficient ≥ 0.6). In contrast, it was observed a wide-spread pattern of fluorescence through the cell with some mitochondrial co-localization (naked-eye observation, Pearson coefficient > 0.2 and < 0.4) with mitotracker for E64D, R98Q, A104T, D149A and A171S missense mutants. Finally, a wide-spread non-mitochondrial distribution of fluorescence without significant co-localization with mitotracker fluorescence (Pearson coefficient ≤ 0.2) was found for DJ-1 WT, L10P, A39S, A171S and K175E. These results suggested that a good approach to study both, protein degradation and subcellular localization, would be to obtain fusion constructs of DJ-1 mutants with fluorescent proteins. To that end, constructs of M26I and L166P fused to the N-terminus of EGFP were produced. The results obtained after transfection in MEFs from DJ-1-null mice (see Supplementary Fig. 3) clearly indicated that the C-terminal tagging of the unstable L166P missense mutant greatly slows down its degradation rate compared to the untagged version (see Fig. 1 for comparison). Similarly, M26I fusion construct was not significantly degraded after 24 h of incubation in the presence of CHX. Furthermore, the fusion proteins did not reproduce the preferential mitochondrial localization observed by indirect immunofluorescence of the untagged M26I and L166P DJ-1 missense mutants (compare Supplementary Figs. 3, 4). In conclusion, the approach with fluorescent fusion constructs to study protein degradation and subcellular localization was discarded.

To get independent evidence of the subcellular localization, biochemical fractionation studies of DJ-1 mutants transfected in DJ-1-null MEFs were performed. Supplementary Fig. 4 shows the results obtained. Cell fractionation studies showed that DJ-1 point mutants M26I, A107P, P158**Δ**, L166P, E163K and L172Q showed significant association with the mitochondrial fraction, while they were also clearly present in the cytoplasmic fraction. This interpretation is further strengthened by comparison to similar fractionation studies with transfected DJ-1 WT and missense point mutants L10P, A39S, E64D, R98Q, A104T, D149A, A171S, K175E and A179T that showed a predominant cytoplasmic distribution and were absent in the mitochondrial fraction (Supplementary Fig. 4).

### Pathway of degradation of DJ-1 unstable missense mutants

**Figure 5:**
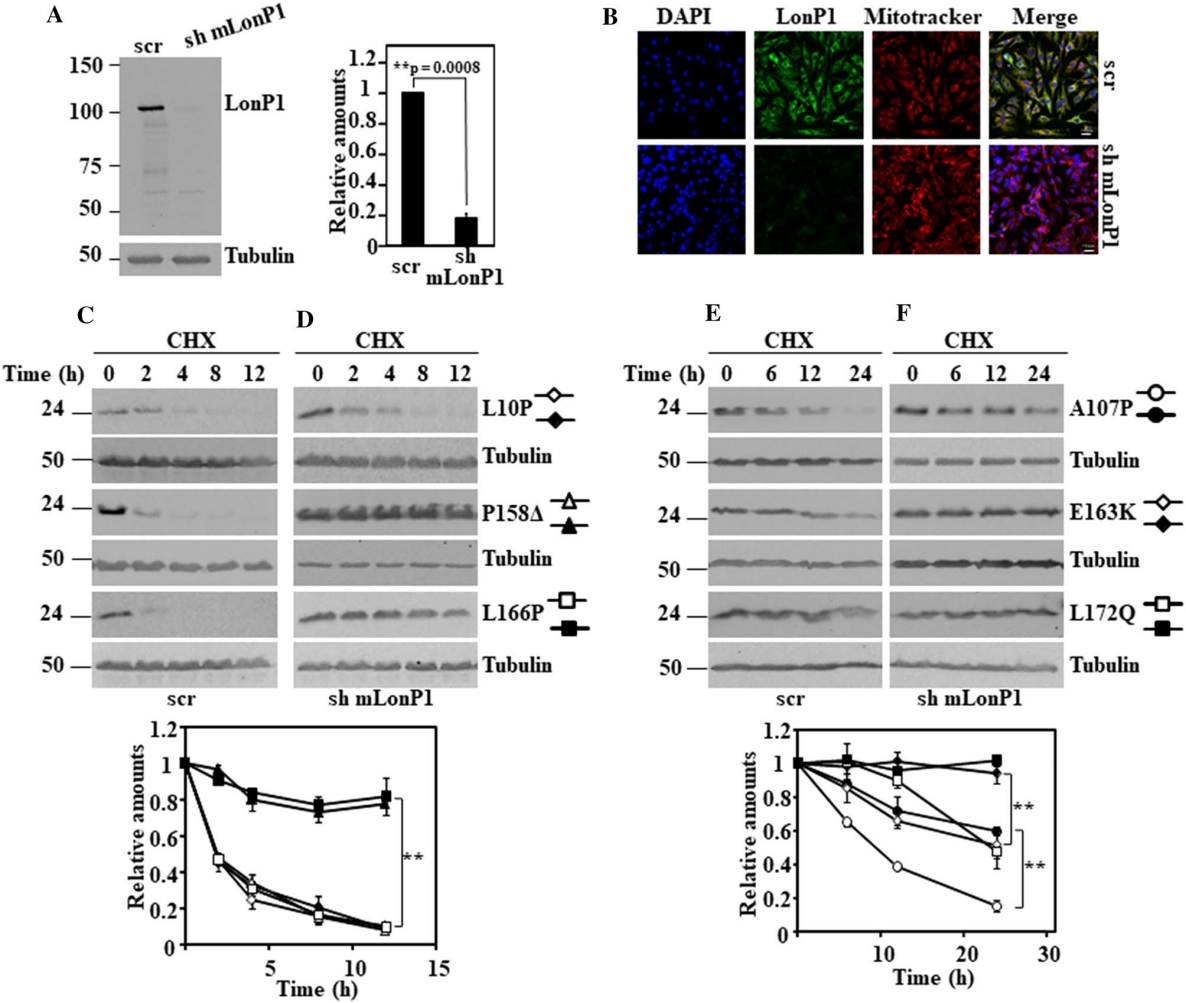
Effect of LonP1 silencing on the degradation of DJ-1 unstable mutants transfected in DJ-1-null MEFs. DJ-1-null MEFs were transduced with either scrambled shRNA (scr) or specific shRNA for mouse LonP1 (sh mLonP1), as described under the Material and Methods section. (**A**) Panel shows a representative immunoblot developed with specific anti-LonP1 antibodies of LonP1 shRNA-mediated knockdown in DJ-1-null MEFs. Quantification is shown in the right graph. (**B**) Confocal fluorescence images of non-target shRNA lentiviral transduced (scr) or LonP1 shRNA lentiviral transduced (sh mLonP1) DJ-1-null MEFs growing under basal conditions and stained with Mitotracker (red), anti-LonP1 specific antibodies (green) and counterstained with DAPI (blue) for nuclei visualization. Scr DJ-1-null MEFs, panels (**C**) and (**E**) or sh mLonP1 DJ-1-null MEFs, panels (**D**) and (**F**), were transiently transfected with hDJ-1 L10P, P158Δ and L166P (**C**,**D**) or hDJ-1 A107P, E163K and L172Q (**E**,**F**) and 48 h after transfection were treated with 25 µg/mL cycloheximide (CHX) for the times indicated. Panels show representative immunoblots with anti-DJ-1 polyclonal antibody of the indicated hDJ-1 missense mutants transfected MEFs. Anti-tubulin antibodies were used as total protein loading control. Quantifications are shown in the graphs below as mean ± s.e.m from three different experiments. Significant differences were found between scr and sh mLonP1 DJ-1-null MEFs transfected with P158Δ at the time points 2 h (**p = 0.0001), 4 h (**p = 0.003), 8 h (**p = 0.0008) and 12 h (**p = 0.009), between scr and sh mLonP1 DJ-1-null MEFs transfected with L166P at the time points 2 h (**p = 0.0002), 4 h (**p = 0.0001), 8 h (**p = 0.0008) and 12 h (**p = 0.002), between scr and sh mLonP1 DJ-1-null MEFs transfected with A107P at the time points 6 h (**p = 0.0008), 12 h (*p = 0.02) and 24 h (**p = 8E−05), between scr and sh mLonP1 DJ-1-null MEFs transfected with E163K at the time points 12 h (**p = 0.009) and 24 h (*p = 0.01) and between scr and sh mLonP1 DJ-1-null MEFs transfected with L172Q at the time point 24 h (**p = 0.001).

The above results seemed to indicate that a mitochondrial pathway could be responsible of the degradation of the unstable DJ-1 mutants. The mitochondrial localization of some unstable DJ-1 mutants and some previous reports describing that DJ-1 may translocate to the mitochondria matrix^63,64^ prompt us to study the possible involvement of mitochondrial matrix proteases in the degradation of DJ-1 missense mutants. The matrix mitochondrial LonP1 protease was examined as a possible candidate, based on it is reported implication in PINK1 degradation (see “Discussion”). *LONP1* is an essential gene in mice^65^, as consequence we were unable to get cells from LonP1 null mice. Consequently, shRNA interference of mouse LonP1 (mLonP1) in DJ-1-null MEFS or N2a cells were used to test this hypothesis. As shown in Fig. 5, the down regulation of LonP1 expression in DJ-1-null MEFs (Fig. 5A immunoblot and Fig. 5B immunofluorescence) resulted in a significant decrease in the degradation rate of unstable DJ-1 missense mutants A107P, P158**Δ**, E163K, L166P and L172Q (Fig. 5C,D), but not of DJ-1 L0P. Similar results were obtained with mLonP1 silencing in N2a cells expressing those missense DJ-1 mutants (Supplementary Fig. 5).

**Figure 6:**
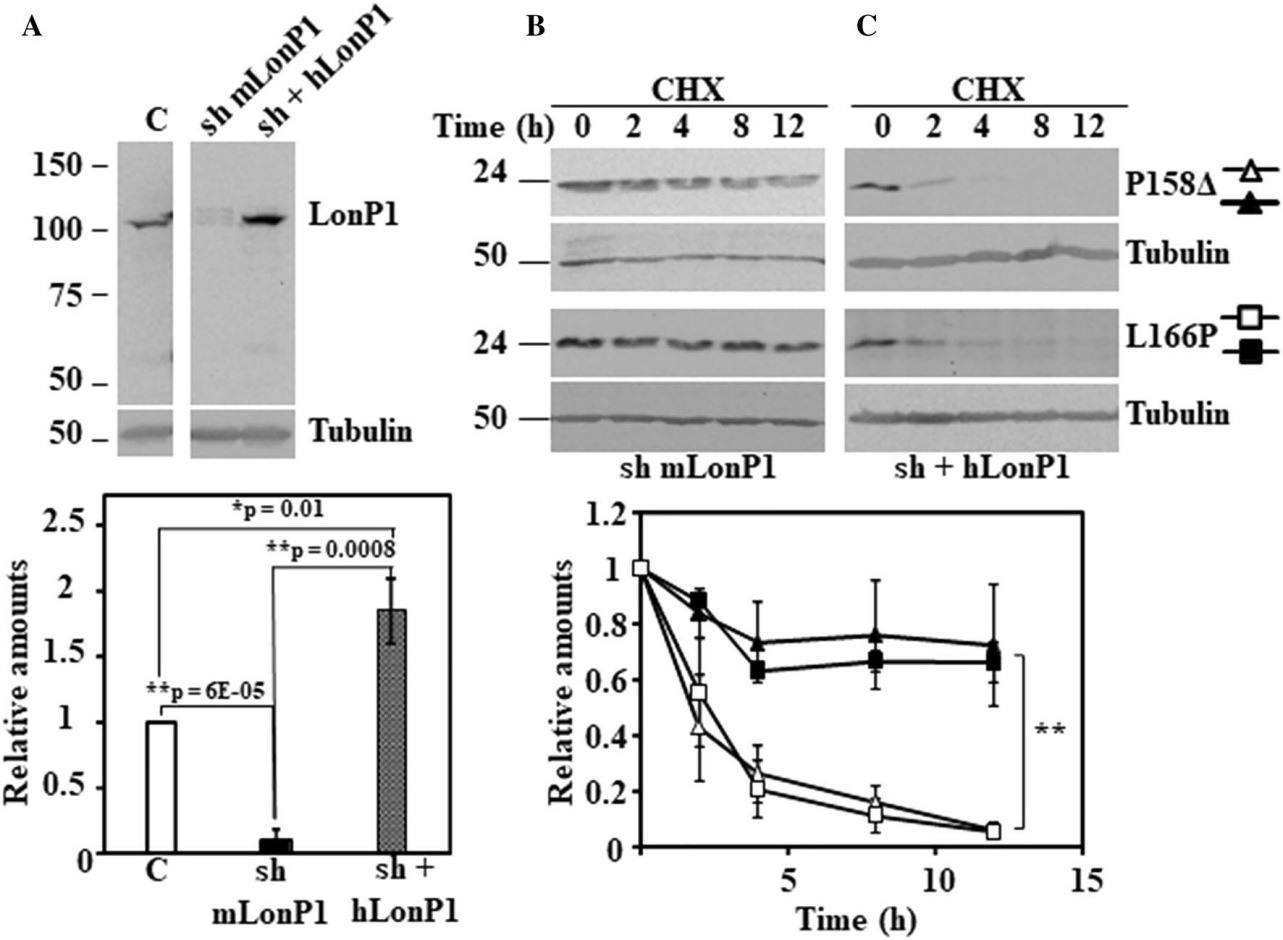
Rescue of the degradation of DJ-1 unstable mutants transfected in LonP1-silenced DJ-1-null MEFs by transfection of human LonP1. LonP1 shRNA-transduced DJ-1-null MEFs (sh mLonP1) were complemented with ectopically expressed human LonP1 (hLonP1). (**A**) Panel shows a representative immunoblot of LonP1 shRNA-mediated knockdown in DJ-1-null MEFs and the recovery of LonP1 protein levels after transfection with hLonP1. Immunoblot was developed with specific antibodies anti-LonP1. Quantification is shown in the right graph. LonP1-silenced DJ-1-null MEFs (sh mLonP1), panel (**B**) and rescued LonP1-silenced DJ-1-null MEFs (sh + hLonP1), panel (**C**), were transiently transfected with hDJ-1 P158Δ or L166P and 48 h after transfection were treated with 25 µg/mL cycloheximide (CHX) for the times indicated. Panels (**B**) and (**C**) show representative immunoblots developed with anti DJ-1 polyclonal antibody of the indicated constructs. Anti-tubulin antibodies were used as total protein loading control. Quantifications are shown in the graphs below as mean ± s.e.m from three different experiments. Significant differences were found between sh mLonP1 and sh + hLonP1 DJ-1-null MEFs transfected with P158Δ at the time points 2 h (*p = 0.01), 4 h (*p = 0.03), 8 h (*p = 0.03) and 12 h (*p = 0.04) and between sh mLonP1 and sh + hLonP1 DJ-1-null MEFs transfected with L166P at the time points 4 h (*p = 0.01), 8 h (**p = 0.001) and 12 h (**p = 0.001).

To check for off-target effects of mLonP1 shRNA, human LonP1 (hLonP1) cDNA was used to rescue the phenotype of mLonP1 silenced cells respect to degradation of two of the unstable DJ-1 mutants. As shown in Fig. 6, over expression of hLonP1 completely rescued the inhibition of degradation of the unstable DJ-1 P158Δ and L166P mutants produced by mLonP1 shRNAs. Taken together, those results clearly allowed the conclusion that LonP1 is the mitochondrial protease is clearly involved in the degradation of unstable DJ-1 mutants A107P, P158Δ, E163K, L166P and L172Q mutants but not in the degradation of DJ-1 L10P mutant.

## Discussion

DJ-1 WT is a stable protein when expressed by transfection in DJ-1-null MEFs, similar to what is found for endogenously expressed DJ-1 in other cells^36,38,43,66–68^. The PD missense mutations A39S, E64D, A104T, D149A, K175E and the polymorphic missense variants R98Q and A171S are as stable as DJ-1WT, both in MEFs from DJ-1-null mice (Fig. 1) and in N2a cells (Supplementary Fig. 1), expressing the endogenous DJ-1 WT. The DJ-1 point mutants L10P, A107P, P158Δ, E163K, L166P and L172Q were degraded with similar half-lives in MEFs from DJ-1-null mice and in N2a cells. The M26I DJ-1 mutant is slowly degraded in MEFs from DJ-1-null mice (see Fig. 1 and Supplementary Table 1), but degradation is not apparent in N2a cells after treatment of cells with CHX for 24 h, as reported previously^43^. These results indicated that the unstable (and untagged) DJ-1 mutants, qualitatively, are unstable even in the presence of the expression of endogenous DJ-1 WT.

Several laboratories (including ourselves) have reported that the missense DJ-1 L166P mutant is degraded by the ubiquitin–proteasome pathway^2,36–43^. Similar conclusions were reported for the degradation of L10P, P158Δ^46,47^ and L172Q^49^ point mutants. Nevertheless, several groups reported that proteasome inhibitors are not able to completely prevent the degradation of the unstable mutants L166P^37,39,42,43,46^, L10P and P158Δ^46^. Furthermore, as shown here, treatment with different inhibitors of the autophagic-lysosomal pathway (NH_4_Cl, leupeptin 3-methyl-adenine and E64) fail to prevent their degradation (Fig. 3). Similar results with those inhibitors have been reported previously for the degradation of DJ-1 L166P missense mutant^37,39,41^.

In the search for alternative pathways of degradation, experiments of subcellular localization of DJ-1 by immunofluorescence and biochemical cell fractionation studies were performed (Fig. 4 and Supplementary Fig. 4, respectively). Those data showed that the DJ-1 point mutants M26I, A107P, P158**Δ**, E163K, L166P and L172Q, but not L10P, are significantly associated with mitochondria. Those results are in agreement with previously published work of the localization of M26I, P158Δ, E163K, L166P and L172Q^2,45,49,63,64,69^; no data are available for A107P point mutant. The widely used experimental approach of using fusion constructs of the missense mutants with fluorescent proteins as reporters for both degradation and subcellular localization was discarded, as the DJ-1 L166P and M26I proteins fused to EGFP (Supplementary Fig. 3) did not meet the simple criteria of similar degradation rates and subcellular localization as their respective untagged DJ-1 missense mutants, another clear demonstration of the caveat of using tagged versions of proteins for studying DJ-1 point mutant behaviour. Similar results for the E63K mutant fused to GFP have been reported^63^.

To study the possible involvement of mitochondria in the degradation of missense DJ-1 mutants, we hypothesized that mitochondrial matrix LonP1 could be implicated. The experimental evidence presented (Fig. 5 and Supplementary Fig. 5) clearly indicated that mitochondrial LonP1 is implicated in the degradation of A107P, P158Δ, E163K, L166P and L172Q. We showed (Fig. 6) that the strong inhibition of their degradation by interrupting the expression of mouse LonP1 can be rescued by overexpression of human LonP1 (whose expression is not suppressed by the action of the specific mouse LonP1-targeting shRNAs), indicating the specificity of the observed inhibitory effects. Formally, it can be argued that rescue by a variant mLonp1 mutant construct insensitive to the shRNA may provide additional, but not essential, confirmation. Furthermore, while certainly hLonp1 is overexpressed in the rescue experiments, the results obtained suggest that those DJ-1 point mutants are also substrates for the hLonP1 protease, as expected. In contrast, there is no effect on L10P degradation. The elucidation of the degradation pathway of this missense mutant requires further investigation.

The localization of DJ-1 in the mitochondria and translocation to mitochondrial matrix have been studied^63,69^. The L10P mutation located in strand 1 prevents homodimer formation while the mutant interacts with DJ-1 WT^46,48^. The L10P mutation may hinder the mitochondrial localization, as that region of strand 1 of the 3D-protein structure^4,5,7^ has been implicated in mitochondrial localization. The DJ-1 E18A point mutant (a-helix 1) localizes to the mitochondria, but the substitution by alanine of the wild -type sequence at leucine (a.a. 7), valine (a.a. 8), isoleucine (a.a. 9) and leucine (a.a. 10, changed to Pro in the missense mutant) within strand 1 prevents mitochondrial localization^69^. The fact that the mutants ΔC15 and M26I (shown also here, Fig. 4) also localized to the mitochondria^63^, further supports the role of the aminoacid sequence of strand 1 and a-helix 1 (aminoacids 5–28) in the N-terminus as the sequence that either prevents the translocation of DJ-1 to mitochondria, or promotes its cytoplasmic retention. But those N-terminal structural elements are clearly insufficient to explain the location to mitochondria, as DJ-1 with point mutations at the C-terminus having an intact N-terminal sequence, also localize to the mitochondria. Clearly further experiments will allow elucidating the structural requirements for the mitochondrial localization and matrix translocation of DJ-1 missense mutants.

Mutations in *LONP1* gene produce the CODAS syndrome with Cerebral, Ocular, Dental, Auricular and Skeletal abnormalities^70,71^, more recently a bi-allelic mutation (c.2282 C > T, (p.Pro761Leu) in *LONP1* results in neurodegeneration with deep hypotonia and muscle weakness, severe intellectual disability and progressive cerebellar atrophy^72^. These observations clearly indicate a relationship of LonP1 with central nervous system abnormalities and neurodegeneration. LonP1 is implicated in the degradation of matrix mitochondrial proteins like aconitase^73^, 5-aminolevulinic acid synthase^74^, TFAM^75^, StAR^76^, complex 1 of the OXPHOS^77^, SDH5^78^ and SDHB^79^ of complex II of OXPHOS and unfolded proteins, like the matrix located OTC Delta used to promote an unfolding protein response (mitUPR) in mitochondria^80,81^. LonP1 also participates in the default degradation in mitochondrial matrix of the PD linked protein kinase encoded by *PINK1/PARK6* gene. Under mitUPR conditions, PINK1 accumulates (increased accumulation was produced with silencing of LonP1) and recruits the PD-linked E3 ligase PARKIN/PARK2 promoting mitochondrial clearance by mitoautophagy^80,82^. Other groups have observed the accumulation of PINK1 upon silencing LonP1 without the need of concomitant induction of mitUPR response^83,84^. PINK1 is initially processed by the mitochondrial processing protease (MPP), other mitochondrial proteases like PARL, ClpPX and AFG3L2 also participate^82^. The processed PINK1, in contrast to the above observations of degradation by LonP1, would be rapidly degraded by the ubiquitin-proteasome pathway^85,86^ after polyubiquitylation^87^. In *Drosophila*, overexpression of DJ-1 rescues the altered phenotype caused by the loss of PINK1, but not of Parkin^88^. Similarly, DJ-1 overexpression also rescues the increased sensitivity of *Substantia Nigra* to MPTP in *PINK1* null mice^89^. Furthermore, the mitochondrial fragmentation phenotype of DJ-1-null cells can be rescued by overexpression of PINK1 or PARKIN^90,91^. Taken together all these results indicate that DJ-1 and PINK1 (PARKIN) probably act in parallel pathways and LonP1 is involved in DJ-1 point mutants and PINK1 degradation, with variable degrees of involvement of the ubiquitin–proteasome pathway and other degradation pathways that may also be subjected to cell-specific determinants.

In conclusion, the PD phenotype presented by patients harbouring homozygous mutations M26I, A107P, P158**Δ**, E163K, L166P and L172Q can be explained by a "loss of function", similar to the effects produced by DJ-1 mutation that resulted in strong down-regulation or absence of DJ-1 mRNA (deletions, CNV and splicing mutations) because of its instability due to its mitochondrial targeting and degradation mainly by matrix mitochondrial LonP1 protease. A similar situation is the case of patients with homozygous DJ-1 L10P mutation, while its main pathway of degradation remains to be determined. In contrast, the other point mutants (A39S, E64D, A104T, D149A, K175E and A179T) and the rare polymorphisms (R98Q, A171S) might not be truly pathogenic (likely sure for the polymorphic variants R98Q, A171S). Another possibility is that their half-life could still be lower than DJ-1 WT, but escape to our detection limits (24 h), requiring the use of quantitative proteomic studies using SILAC pulse-chase experiments and MS for its determination (far away from the scope and budget of our work). By those methods the DJ-1 WT protein shows a very long half-life (t1/2), from t1/2 = 187 h in mouse NIH3T3 cells^66^ to t1/2 = 199 to 299 h as determine in vivo in mouse heart^92^. Accordingly, the pathogenetic mechanism for A39S, E64D, R98Q, A104T, D149A, A171S, K175E and A179T remains to be determined.

## Materials and methods

### Plasmid constructs

The vectors for expression of untagged human wild type DJ-1 (hDJ-1 WT) and missense mutants M26I, R98Q, A104T, D149A and L166P have been previously described^43^. The mutant L10P was obtained by PCR (4 min, 97 °C; 30 cycles of 45 s, 95 °C; 1 min, 61 °C and 1.5 min, 72 °C, and a final polymerization cycle of 15 min, 72 °C) with the appropriate oligonucleotides, forward BamH1-hDJ-1 L10P: 5′ GAGCGGATCCATGGCTTCCAAGAGGGCTCTGGTCATCCCGGCTAAAGGAGCAGAGG 3′ and reverse XhoI-hDJ-1 L10P: 5′ GCGCCTCGAGCTAGTCTTTAAGAACAAGTGGAGCC 3′ and amplified DNA was subcloned into pCMV-Sport6 vector for expression in eukaryotes, as described^43^.

The mutations A39S, E64D, A107P, P158Δ, E163K, A171S, K175E and A179T of hDJ-1 were introduced by PCR (initial 4 min, 97 °C; 30 cycles of 45 s, 95 °C; 1 min, 61 °C and 10 min, 72 °C, and a final polymerization cycle of 15 min, 72 °C) from hDJ-1 WT inserted in pCMV-Sport6 using the method of QuickChange Site-Directed Mutagenesis Kit (Statagene, La Jolla, California, USA) with the following oligonucleotides: forward hDJ-1 A39S: 5′CCGTTGCAGGCCTGTCTGGAAAAGACCCAGTAC 3′ and reverse hDJ-1 A39S 5′GTACTGGGTCTTTTCCAGACAGGCCTGCAACGG 3′; forward hDJ-1 E64D: 5′GCAAAAAAAGAGGGACCATTTGATGTGGTGGTTC 3′, and reverse hDJ-1 E64D: 5′GAACCACCACATCAAATGGTCCCTCTTTTTTTGC 3′; forward hDJ-1 A107P: 5′GATAGCCGCCATCTGTCCAGGTCCTACTGCTCTG 3′ and reverse hDJ-1 A107P: 5′CAGAGCAGTAGGACCTGGACAGATGGCGGCTATC 3′; forward hDJ-1 P158Δ: 5′CTGATTCTTACAAGCCGGGGTGGGACCAGCTTCGAG 3′ and reverse hDJ-1 P158Δ: 5′CTCGAAGCTGGTCCCACCCCGGCTTGTAAGAATCAG 3′; forward hDJ-1 E163K: 5′GGACCAGCTTTAAGTTTGCGCTTGC 3′ and reverse hDJ-1 E163K: 5′GCAAGCGCAAACTTAAAGCTGGTCC 3′; forward hDJ-1 A171S 5′GCAATTGTTGAATCCCTGAATGGCAAGGAGG 3′ and reverse hDJ-1 A171S: 5′CCTCCTTGCCATTCAGGGATTCAACAATTGC 3′; forward hDJ-1 K175E: 5′GCCCTGAATGGCGAGGAGGTGGCGGCTCAGG 3′ and reverse hDJ-1 K175E: 5′CTTGAGCCGCCACCTCCTCGCCATTCAGGGC 3′; forward hDJ-1 A179T: 5′GCAAGGAGGTGGCGACTCAAGTGAAGGC 3′ and reverse hDJ-1 A179T 5′GCCTTCACTTGAGTCGCCACCTCCTTGC 3′. The hDJ-1 L172Q mutant was synthesized by GenScript (Piscataway, New Jersey, USA).

The EGFP fusion protein constructs of hDJ-1 M26I and L166P were obtained by gene synthesis of hDJ-1 cDNA missense mutants including restriction sites 5′ for XhoI and 3′ for AgeI and subcloning into the XhoI-AgeI sites of the pEGFP-N1 mammalian expression vector (GenScript).

Mouse LonP1 shRNA (RMM4534-NM_028782) and the corresponding scramble control shRNA cloned in the lentiviral vector pLKO.1 puro^65^ were provided by Dr. Carlos López-Otín, Departamento de Bioquímica y Biología Molecular, Universidad de Oviedo, Spain. For rescue experiments in mouse LonP1 silenced cells, human LonP1 cDNA (clone MGC:1498 IMAGE:3350958) was obtained from the pOTB7 construct by digestion with EcoR1 and XhoI and subcloned into pcDNA 3.1 vector, previously digested with the same restriction enzymes. The DNA sequence of all constructs was verified by Sanger sequencing in an ABI Prism 3130XL (Applied Biosystems, Foster City, California, USA).

### Cell culture, transfections and treatments

Mouse embryonic fibroblasts (MEFs) from DJ-1-null mice were provided by Dr. Jie Shen, Center for Neurologic Diseases, Brigham and Women's Hospital and Harvard Medical School, Boston, USA and grown in Dulbecco’s modified Eagle’s medium with low glucose (1 g/L) and supplemented with 15% fetal bovine serum and 50 µg/mL gentamicin. DJ-1-null MEFs (3 × 10^6^) were transiently transfected with 6 µg of the indicated DJ-1 constructs by nucleofection (Nucleofector II program T-016, Amaxa Nucleofector Technology, Basel, Switzerland) by using an in-house made transfection buffer (150 mM KH_2_PO_4_, 24 mM NaHCO_3_, 4 mM D-glucose, 6 mM ATP and 12 mM MgCl_2_). Mouse neuroblastoma N2a cells were cultured in Dulbecco’s modified Eagle’s medium with 4.5 g/L glucose supplemented with 10% fetal bovine serum and 50 µg/mL gentamicin. N2a cells (3 × 10^5^) were transiently transfected using Lipofectamine 2000 reagent (Thermo Fisher Scientific, Waltham, Massachusetts, USA) according to the manufacturer's instructions.

Transfected cells were grown for 48 h and then protein degradation was studied by treatment of cells with 25 µg/mL cycloheximide (CHX) for the times indicated. At the dose of CHX indicated, cell viability (MEFs and N2a, trypan blue exclusion) was > 95% after 24 h of incubation. For some experiments, transiently transfected DJ-1-null MEFs were kept in complete medium or treated with 25 µg/mL CHX in the absence or the presence of 10 µM MG-132, 20 mM NH_4_Cl, 20 mM NH_4_Cl plus 50 µM leupeptin (Leu) or 10 mM 3-methyl adenine (3MA) plus 5 µM E64 for 12 or 24 h, as indicated. Cells were then washed three times with cold phosphate-buffered saline (PBS), pelleted by centrifugation and used for the obtention of total cell extracts.

### Lentiviral production

For lentiviral production, 3.5 × 10^6^ HEK293T cells (cultured in Dulbecco’s modified Eagle’s medium with 4.5 g/L glucose supplemented with 10% fetal bovine serum and 50 µg/mL gentamicin) were seeded in 20 mm × 100 mm culture dishes and the next day transfected with 3.2 µg of the packaging plasmid psPAX2, 1.6 µg of the envelope plasmid pM2.G and 5 µg of the corresponding lentiviral transfer plasmid, scramble control shRNA or mouse LonP1 shRNA in pLKO.1 puro plasmid^65^, by using Lipofectamine 2000 reagent according to the manufacturer's protocol (Thermo Fisher Scientific, Waltham, Massachusetts, USA). The culture medium was removed 24 h after transfection and complete fresh medium was added. Lentiviral particles were harvested 48 h after transfection, again fresh culture medium was added and 72 h after transfection a second harvest of lentiviral particles was collected. The collected culture media were centrifuged at 200 × *g* for 5 min, filtered-sterilized through 0.45 µm filters (Millipore, Darmstadt, Germany), aliquoted and stored frozen at − 80 °C until used for further experiments.

### Cell transduction for LonP1 silencing and rescue experiments

DJ-1-null MEFs (1.3 × 10^5^ per well) or N2a cells (3 × 10^5^ per well) were seeded in 6-well culture plates and incubated with 700 µL of filtered lentivirus-containing medium (scramble control shRNA or mouse LonP1 shRNA) in the presence of 8 µg/mL polybrene to a final volume of 1.4 mL per well. Culture media was replaced 24 h after infection, 48 h after infection transduced cells were selected by addition of 8 µg/mL puromycin to the culture media for four days. Puromycin-resistant cells were transiently transfected with the indicated human DJ-1 constructs and treated with CHX for studying protein degradation, as described above.

For rescue experiments in mouse LonP1 silenced MEFs, puromycin-resistant MEFs were transiently transfected with human LonP1, selected with culture medium containing 250 µg/mL zeocin for four days, transiently transfected with the indicated human DJ-1 constructs and treated with CHX for studying protein degradation, as described above.

### Cell fractionation studies

For biochemical subcellular fractionation experiments, DJ-1-null MEFs were transfected with the indicated untagged human DJ-1 constructs and 48 h after transfection cells were washed with cold PBS for three times, pelleted by centrifugation at 110 × *g* for 5 min at 4 °C, suspended in lysis buffer (20 mM HEPES pH 7.4, 250 mM sucrose, 5 mM MgCl_2_, 10 mM KCl, 1 mM EDTA, 1 mM EGTA, 25 mM NaF, 1 mM orthovanadate, 10 µM leupeptin, 1 µg/mL pepstatin and 1 mM PMSF) and incubated on ice for 10 min, with thoroughly pipetting up and down the suspension each 2 min. Next, the cell suspension was incubated for 5 min at room temperature with lysis buffer containing digitonin (to a final concentration of 50 µg/mL), cell lysis was verified by Trypan blue staining. Cell suspensions were then transfered to ice and centrifuged at 1000 × *g* for 5 min at 4 °C to remove nuclei, debris and non lysed cells. The supernatant was used as the total fraction (input) and was further centrifuged at 15,000 × *g* for 15 min at 4 °C. The supernatant was used as the cytosolic fraction whereas the pellet, containing mitochondria, was washed twice with 200 µL of lysis buffer without digitonin and centrifuged at 15,000 × *g* for 15 min at 4 °C. After the last wash, the pellet was resuspended in a final volume identical to the volume of the cytosolic fraction with a buffer containing 10 mM HEPES pH 7.4, 10 mM KCl, 1 mM DTT, 0.6% NP-40, 1 mM EDTA, 1 mM EGTA, 25 mM NaF, 1 mM orthovanadate, 10 µM leupeptin, 1 µg/mL pepstatin and 1 mM PMSF, vortexed for 10 s and centrifuged for 30 s at 15,000 × *g* at 4 °C. The supernatant of this centrifugation was used as the mitochondrial fraction. Samples from the total input, and mitochondrial and cytoplasmic fractions were loaded onto SDS-PAGE and analyzed by Western and immunoblot, as described below.

### Western immunoblotting

After the treatments, cells were directly lysed in SDS-buffer (62.5 mM Tris–HCl pH 6.8, 2% SDS, 20% glycerol, 10 µM leupeptin, 1 µg/mL pepstatin and 1 mM PMSF). Cell extracts were sonicated for 10 min on ice, centrifuged at 15,000 × *g* for 10 min and supernatants used to measure total protein concentration with BCA protein assay kit (Thermo Scientific-Pierce, Waltham, Massachusetts, USA). Equal amounts of total protein were loaded onto 10% or 14% SDS-PAGE for Western transfer to PVDF membranes and immunoblotting.

Immunoblots were probed with mouse anti-DJ-1 monoclonal antibody (1:1000, MBL, Woburn, Massachusetts, USA, Clone 3E8); rabbit anti-DJ-1 polyclonal antibody (1:1000, Abcam, Cambridge, UK, ab18257); rabbit anti-LonP1 polyclonal antibody (1:1000, Abcam ab103809) and rabbit anti-Tim23 polyclonal antibody (1:1000, Abcam ab230253). Mouse α-Tubulin (1:10,000, DM1A, Sigma, Darmstadt, Germany) monoclonal antibody was used as loading control. The blots were developed with a peroxidase-labeled goat anti-mouse or anti-rabbit secondary antibody (1:5000, Biorad, Hercules, California, USA) and chemiluminiscence detection MF-ChemiBIS 3.2 (DNR Bio-Imaging Systems, Neve Yamin, Israel). Blots were analyzed by quantitative densitometry using Totallab TL100 software (version 1.0, TotalLab Ltd., Newcastle upon Tyne, UK) and protein levels were normalized respect to tubulin.

### Immunofluorescence, confocal microscopy and image analysis

Cells grown on coverslips in 24-well plates were stained for 45 min with 250 nM MitoTracker Red (Invitrogen). The coverslips were then washed with cold PBS three times, fixed with 4% paraformaldehyde in PBS for 20 min at room temperature, permeabilized in PBS with 0.1% Triton X-100 and blocked with PBS and 3% BSA for 1 h at room temperature. The coverslips were processed for indirect immunofluorescence by incubation with rabbit anti-DJ-1 polyclonal antibody (1:200, Abcam) or rabbit anti-LonP1 polyclonal antibody (1:100, Abcam) for 3 h at room temperature, washed with PBS three times for 10 min followed by incubation with Alexa-488 or Alexa-647 fluorescent-labelled secondary antibodies (1:1000) in blocking buffer for 1 h. Next, coverslips were washed with PBS three times for 10 min and 1 μg/mL DAPI (4′,6-diamidino-2-phenylindole) was included for nuclear counterstaining in the second of the washing steps. Coverslips with transfected cells with EGFP fusion protein constructs of DJ-1 were processed for direct fluorescence visualization by incubation with 1 μg/mL DAPI for 5 min. Coverslips were mounted with Prolong Gold antifade reagent (Invitrogen) for confocal microscopy observation in a laser scanning microscope (Leica TCS SP5, Wetzlar, Germany).

To quantify the co-localization between MitoTracker fluorescence (red channel) and the immunofluorescence of transfected human DJ-1 constructs (green channel), single planes along the z-axis of the confocal fluorescence images obtained were analysed with the Image Correlation Analysis (ICA) plugin of ImageJ software^93^. After background subtraction, a single cell was defined as a region of interest (ROI) and quantitative co-localization analysis of the pixels for both channels (red and green) was measured using Pearson’s correlation coefficient the hDJ-1/MitoTracker. Data are presented as mean ± s.e.m from at least 20 individual cells from two different experiments.

### Statistical analysis

Quantitative data are reported as means ± s.e.m from three different experiments. When indicated, values are expressed as mean ± upper and lower values from two different experiments. Statistical significance between groups was calculated using a two-tailored Student’s t-test.

## Supplementary Information

Supplementary Information.
