# Supplementary material for: Mitochondrial LonP1 protease is implicated in the degradation of unstable Parkinson's disease-associated DJ-1/PARK 7 missense mutants
Source: Sci Rep. 2021 Apr 1;11:7320. doi: 10.1038/s41598-021-86847-2 (PMC8016953; doi:10.1038/s41598-021-86847-2)
Supplement: Supplementary file 1 — Supplementary Information. [file 41598_2021_86847_MOESM1_ESM.pdf]

Supplementary Materials

Mitochondrial LonP1 protease is implicated in the degradation of unstable Parkinson's disease-associated *DJ-1/PARK7* missense mutants.

Raúl Sánchez-Lanzas[1,2] and José G. Castaño[1,2]*
[1] Departamento de Bioquímica, [2] Instituto de Investigaciones Biomédicas “Alberto Sols”, UAM-CSIC. Facultad de Medicina UAM. 28029 Madrid. Spain.

* To whom all correspondence should be addressed at: Departamento de Bioquímica e Instituto de Investigaciones Biomédicas "Alberto Sols". UAM-CSIC. Facultad de Medicina de la Universidad Autónoma de Madrid. 28029 Madrid. Spain. FAX: 34-91-585-4401. Email: joseg.castano@uam.es

.

	**DJ-1 null MEFs**	
	**T (1/2) h mean**	**SEM**
**L10P**	**2.6**	**0.35**
**M26I**	**49.79**	**2.05**
**A107P**	**9.70**	**0.45**
**P158Δ**	**3.79**	**0.39**
**E163K**	**20.57**	**2.77**
**L166P**	**3.15**	**0.10**
**L172Q**	**15.20**	**2.54**

Supplementary Table 1. Half-life of unstable human DJ-1 missense mutants transfected in DJ-1 null MEFs. Reported values of the half-life (T ½) of these mutants in DJ-1 null MEFs are from the data of the experiments presented in Fig. 1. Values are expressed in hours (h) as mean ± s.e.m. from three different experiments.

Legends to Supplementary Figures

Legend to Supplementary Fig. 1

Degradation of human wild type DJ-1 and missense mutants in N2a transfected cells. N2a cells were transiently transfected with the indicated untagged human DJ-1 (hDJ-1) constructs and 48h after transfection were treated with 25µg/mL cycloheximide (CHX) for the times indicated. A, panels show representative immunoblots with anti-DJ-1 monoclonal antibody of hDJ-1 wild type (WT), A39S, A171S, K175E and A179T transfected cells. B, panels show representative immunoblots with anti-DJ-1 monoclonal antibody of hDJ-1 L10P, A107P, P158Δ, E163K, L166P and L172Q transfected N2a cells. Anti-tubulin antibodies were used as total protein loading control. Below are shown the graphs of quantification of the corresponding immunoblots. Data are mean ± s.e.m from three different experiments. Significant differences were found between hDJ-1 WT and hDJ-1 L10P at time points 6 hours (**p = 3E-06), 12 hours (**p = 3E-06) and 24 hours (**p = 8E-06), between hDJ-1 WT and hDJ-1 A107P at time points 6 hours (**p = 0.0004), 12 hours (**p = 3E-05) and 24 hours (**p = 1E-06), between hDJ-1 WT and hDJ-1 P158Δ at time points 6 hours (**p = 5E-06), 12 hours (**p = 5E-06) and 24 hours (**p = 1E-06), between hDJ-1 WT and hDJ-1 E163K at time points 6 hours (*p = 0.01), 12 hours (**p = 0.001) and 24 hours (*p = 0.02), between hDJ-1 WT and hDJ-1 L166P at time points 6 hours (**p = 3E-06), 12 hours (**p = 3E-06) and 24 hours (**p = 0.0003) and between hDJ-1 WT and hDJ-1 L172Q at time points 12 hours (*p = 0.04) and 24 hours (*p = 0.04). n.s., not significant.

Legend to Supplementary Fig. 2

Analysis of the co-localization of Mitotracker fluorescence and immunofluorescence of wild type DJ-1 and missense mutants in transfected DJ-1-null MEFs. DJ-1-null MEFs were transfected with the indicated untagged human DJ-1 constructs, stained with Mitotracker (red channel) and processed for immunofluorescence with anti-DJ-1 (green channel) polyclonal specific antibodies. Graph shows the quantitative co-localization analysis between DJ-1 and Mitotracker performed in the confocal fluorescence images obtained as measured by the mean of the DJ-1 / Mitotracker Pearson's correlation coefficient. Data are presented as mean ± s.e.m from at least 20 individual cells.

Legend to Supplementary Fig. 3

Subcellular localization by direct fluorescence and degradation of M26I and L166P DJ-1 - EGFP fusion constructs transfected in DJ-1-null MEFs. A, confocal fluorescence localization of M26I-EGFP or L166P-EGFP in transfected DJ-1-null MEFs growing under basal conditions, stained with Mitotracker (red), analysed by direct fluorescence of EGFP fusion protein (green) and counterstained for nuclei visualization with DAPI (blue). M26I-EGFP or L166P-EGFP transfected DJ-1-null MEFs were treated with 25μg/mL cycloheximide (CHX) for the times indicated and total cell lysates were analysed by Western and immunoblot. B, panels show representative immunoblots with anti-DJ-1 polyclonal antibody of M26I-EGFP and L166P-EGFP transfected cells. Anti-tubulin antibodies were used as total protein loading controls. Quantification of the corresponding immunoblots is shown in the graphs of the right. Data are expressed as mean ± upper and lower limit from two different experiments.

Legend to Supplementary Fig. 4

Biochemical cell fractionation studies of human wild type DJ-1 and missense mutants transfected in DJ-1-null MEFs. DJ-1-null MEFs were transiently transfected with the indicated untagged human DJ-1 constructs and 48h after transfection were processed for subcellular fractionation, as described under the Material and Methods section. Proteins from whole cell lysates (Input), cytoplasmic fraction (Cyt) and mitochondrial fraction (Mit) were analysed by Western and immunoblot with the indicated specific antibodies: anti-DJ-1 polyclonal antibody, anti-Tim23 as a mitochondrial marker and anti-tubulin as a cytoplasmic marker. Panels show representative immunoblots from two different experiments.

Legend to Supplementary Fig. 5

Effect of LonP silencing on the degradation of unstable DJ-1 mutants transfected in N2a cells. N2a cells were transduced with either non-target shRNA (scr) or LonP1 shRNA (sh mLonP1) as described under the material and method section. A, panel shows a representative immunoblot of LonP1 shRNA-mediated knockdown in N2a cells developed with specific antibodies anti-LonP1. Quantification is shown in the right graph. B, Non-target shRNA lentiviral transduced (scr) or LonP1 shRNA lentiviral

transduced (sh mLonP1) N2a cells were transiently transfected with the indicated untagged hDJ-1 constructs and 48 hours after transfection were treated with 25μg/mL cycloheximide (CHX) for the times indicated. Panels B (scr) and C (sh mLonP1) show representative immunoblots with anti-DJ-1 monoclonal antibody of hDJ-1 L10P, P158Δ and L166P transfected N2a cells. Panels D (scr) and E (sh mLonP1) show representative immunoblots with anti-DJ-1 monoclonal antibody of hDJ-1 A107P and E163K transfected cells. Anti-tubulin antibodies were used as total protein loading control. Quantifications are shown in the graphs below as mean ± s.e.m from three different experiments. Significant differences were found between non-target shRNA lntiviral transduced (scr) and LonP1 lentiviral transduced (sh mLonP1) N2a cells (**p = 1E-05), between scr and sh mLonP1 N2a cells transfected with P158Δ at the time points 2 hours (**p = 0.002), 4 hours (**p = 3E-07), 8 hours (**p = 0.002) and 12 hours (*p = 0.01), between scr and sh mLonP1 N2a cells transfected with L166P at the time points 2 hours (**p = 0.006), 4 hours (**p = 0.009), 8 hours (*p = 0.01) and 12 hours (**p = 0.008), between scr and sh mLonP1 N2a cells transfected with A107P at the time points 6 hours (p = 0.03), 12 hours (**p = 0.0008) and 24 hours (**p = 0.002), between scr and sh mLonP1 N2a cells transfected with E163K at the time points 12 hours (*p = 0.04) and 24 hours (**p = 0.005) and between scr and sh mLonP1 N2a cells transfected with L172Q at the time points 12 hours (*p = 0.04) and 24 hours (*p = 0.01).

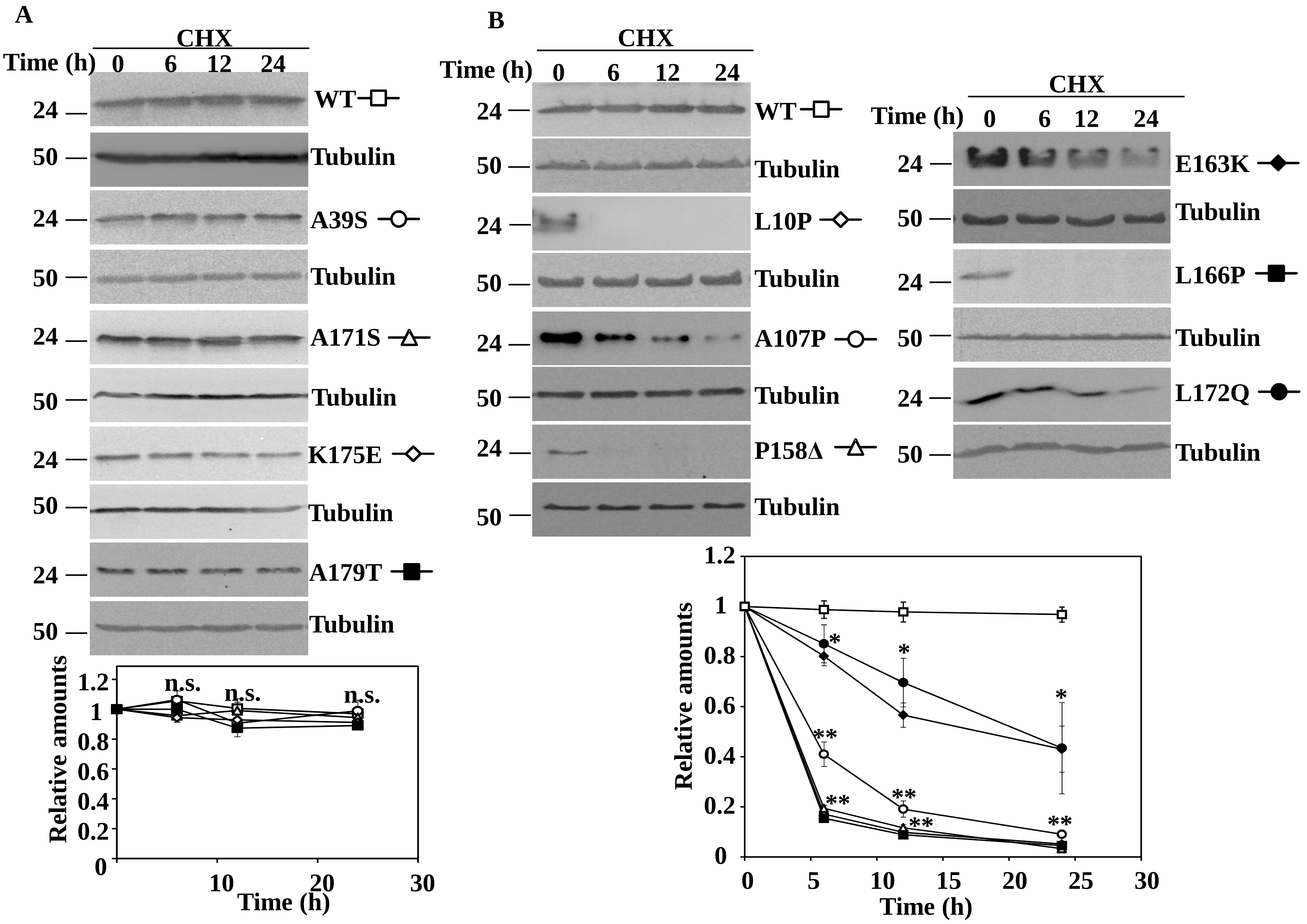

Supplementary Figure 1

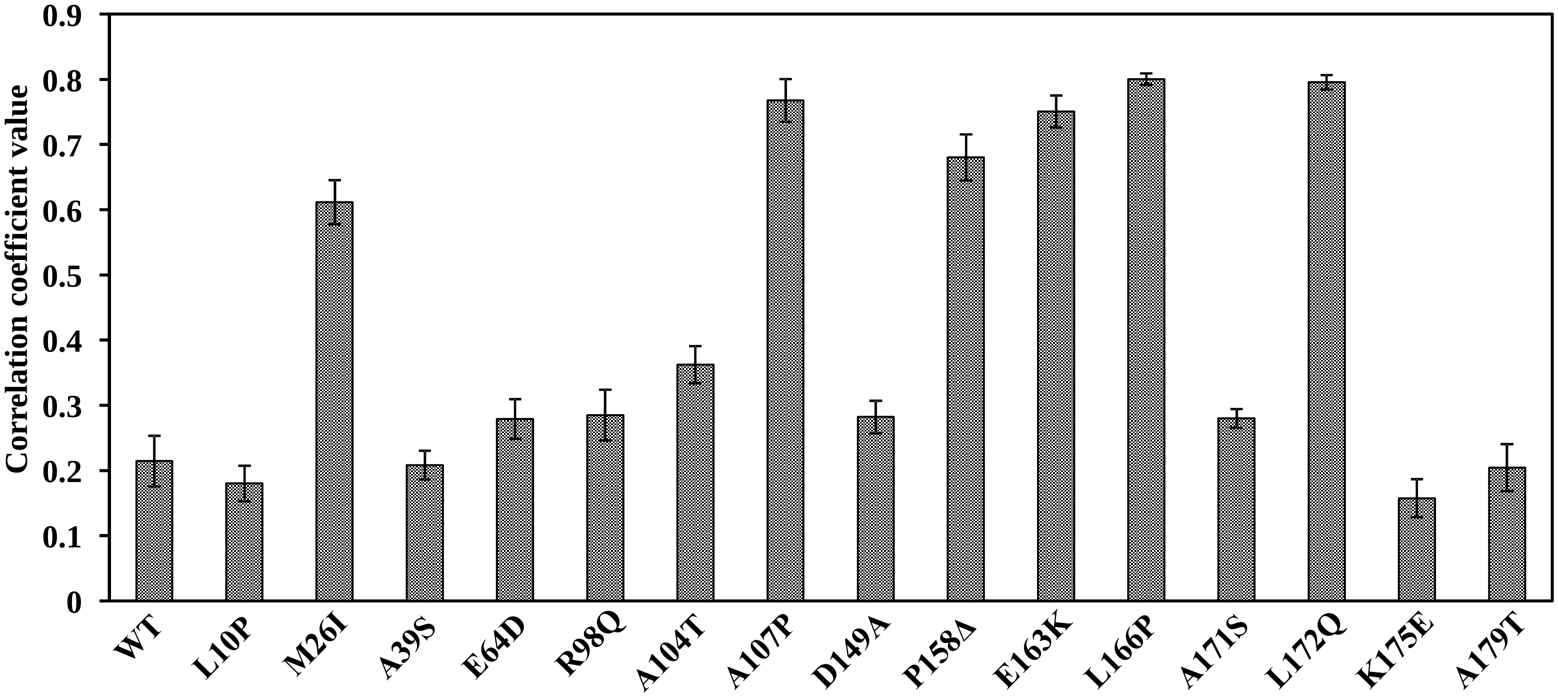

Supplementary Figure 2

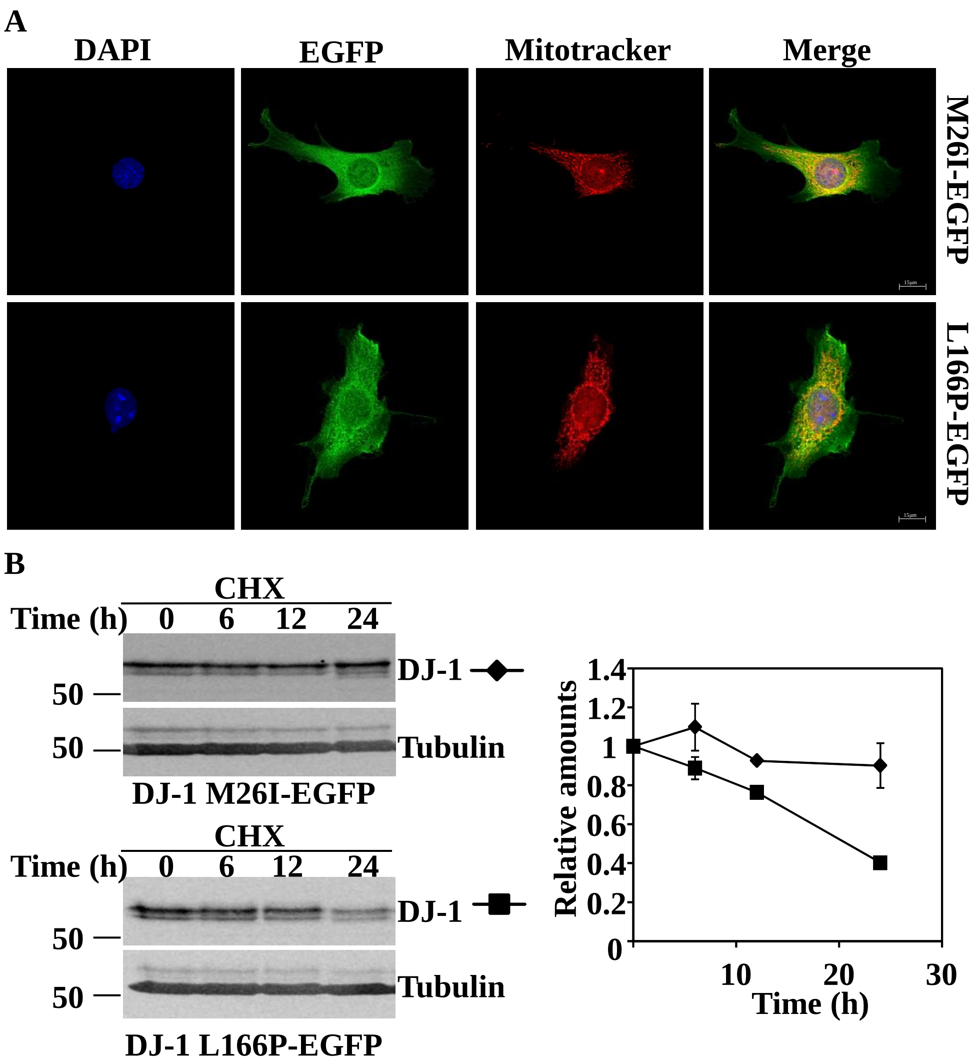

Supplementary Figure 3

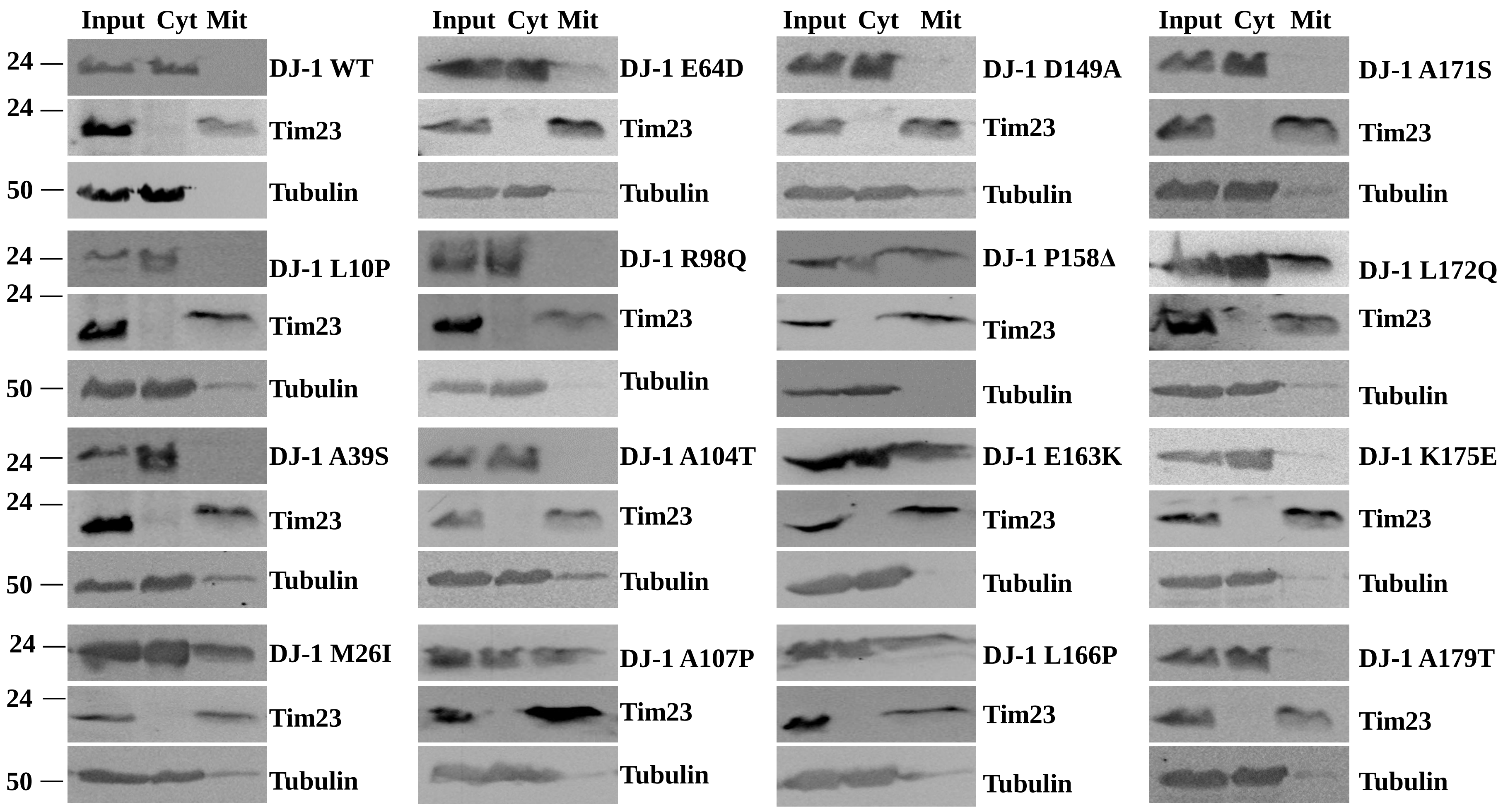

Supplementary Figure 4

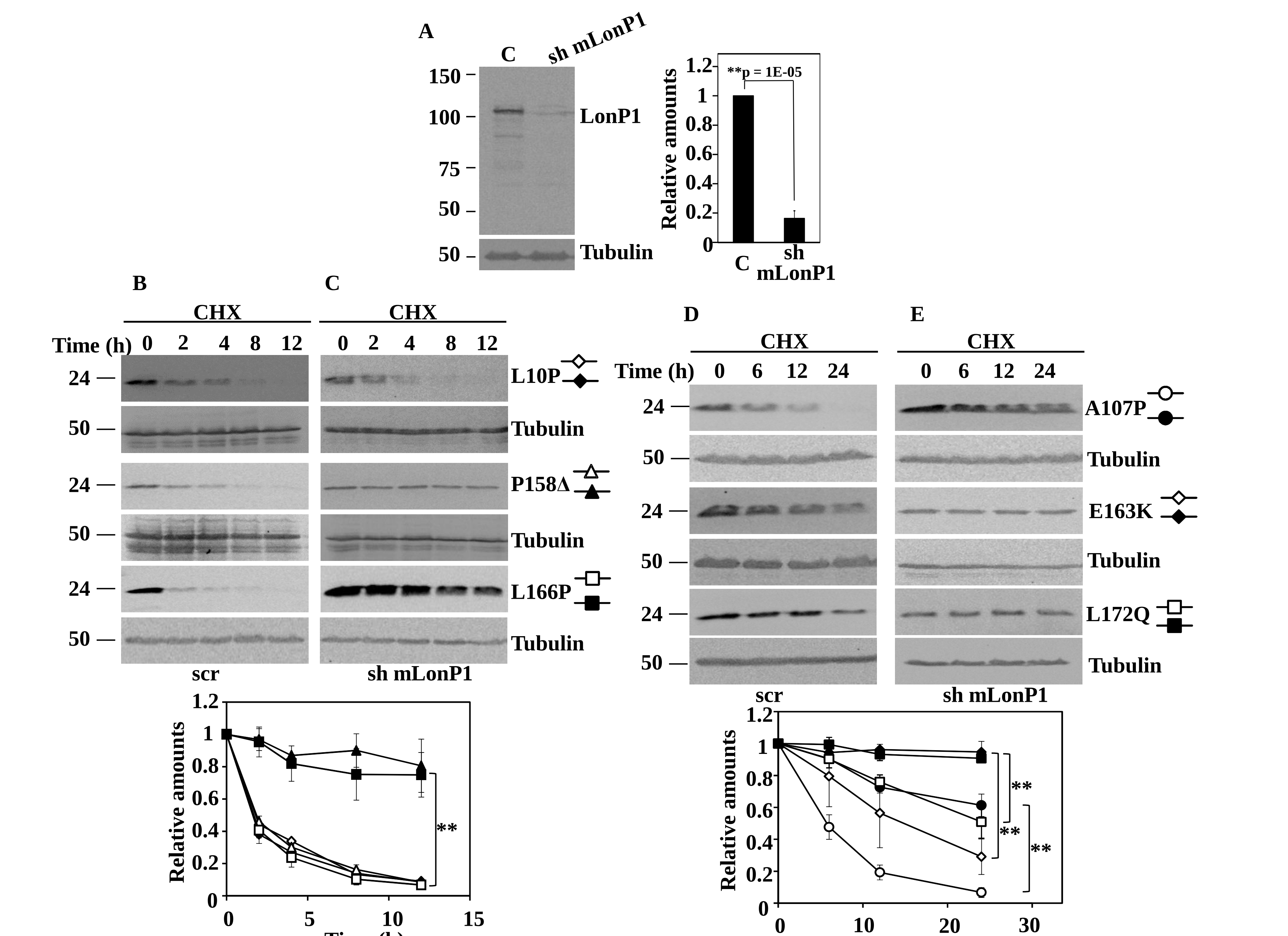

Supplementary Figure 5

Title: Mitochondrial LonP1 protease is implicated in the degradation of unstable Parkinson's disease-associated *DJ-1/PARK 7* missense mutants.

Authors: Raúl Sánchez-Lanzas and José G. Castaño

Uncropped full-length blots corresponding to the immunoblots used in the figures of the paper.

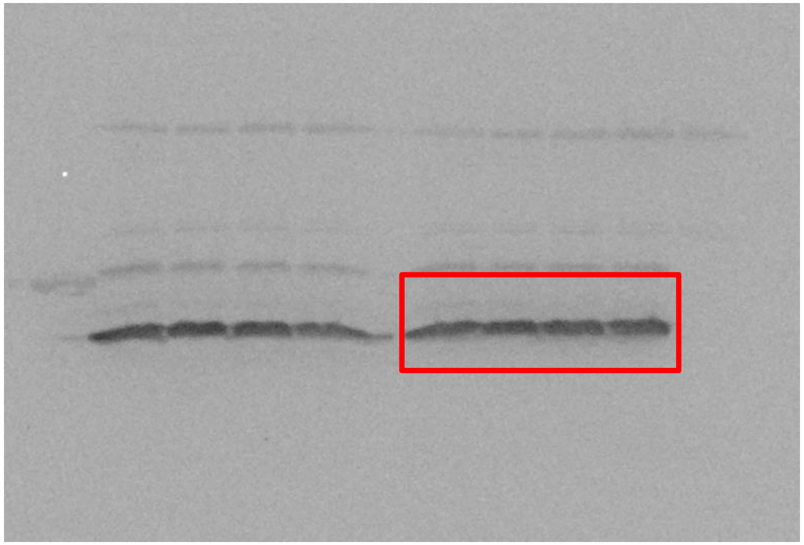

DJ-1 WT

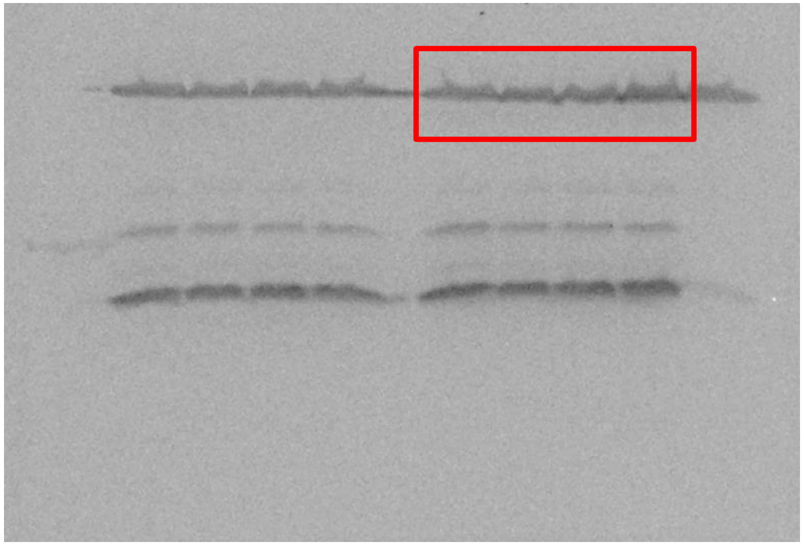

Tubulin

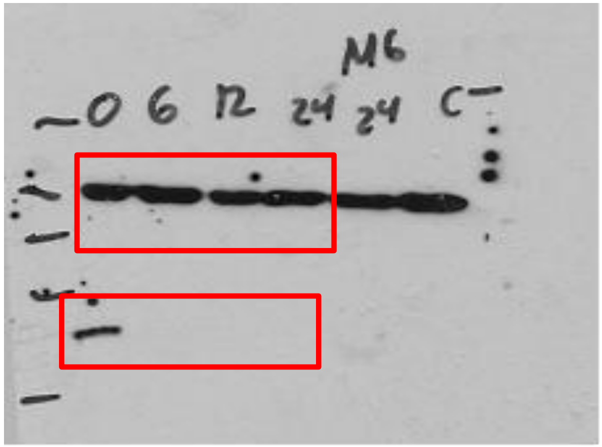

Tubulin

DJ-1 L10P

Figure 1

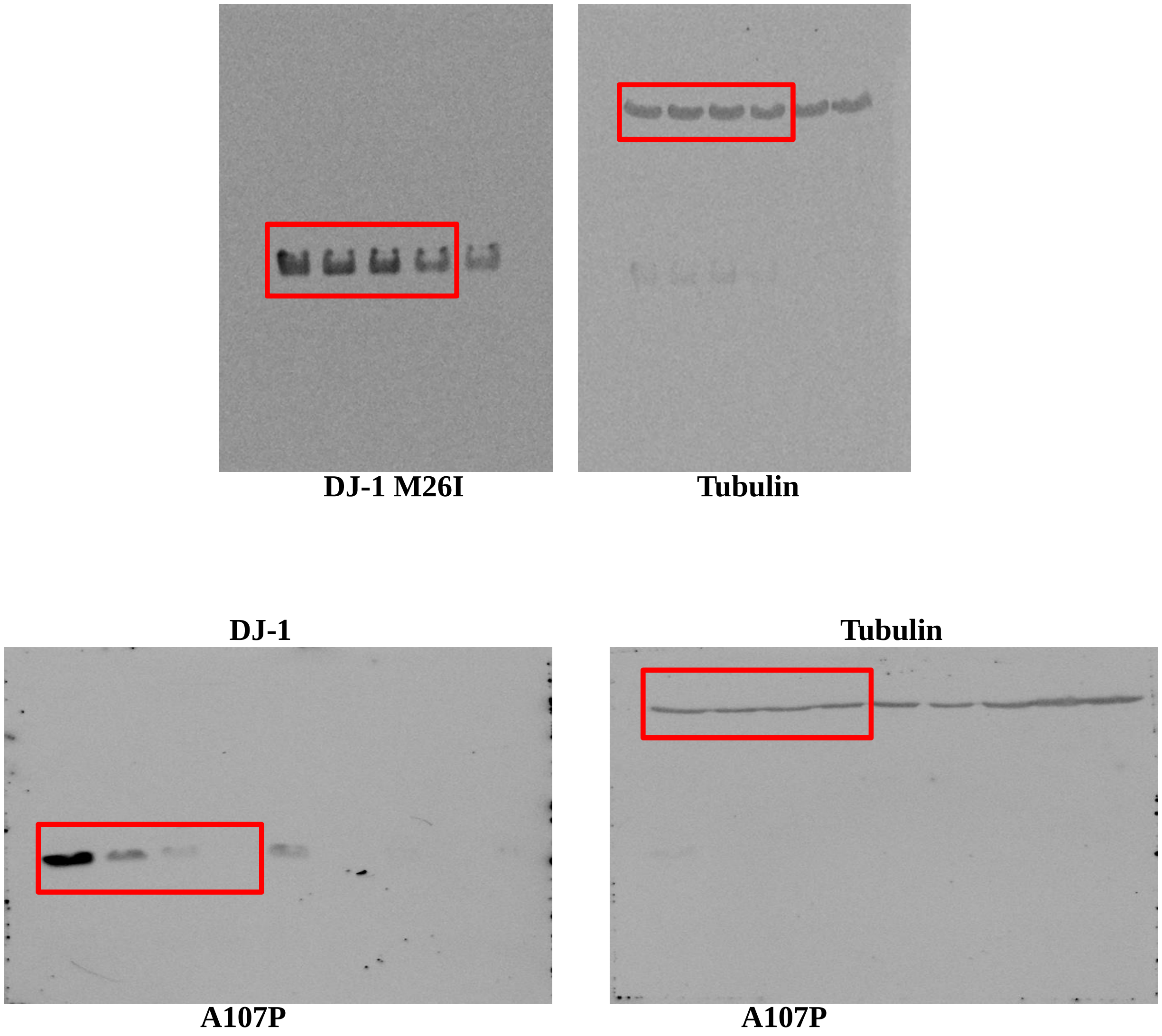

Figure 1

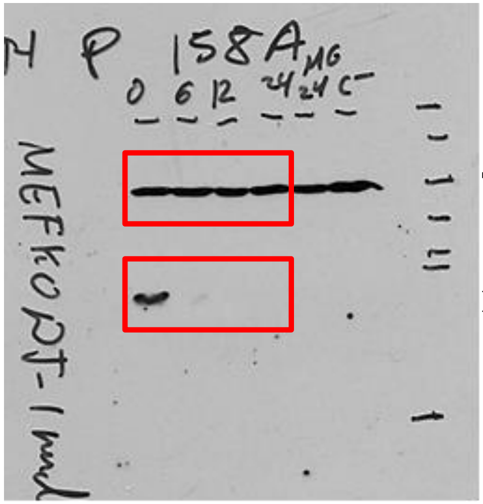

Tubulin

DJ-1 P158Δ

DJ-1

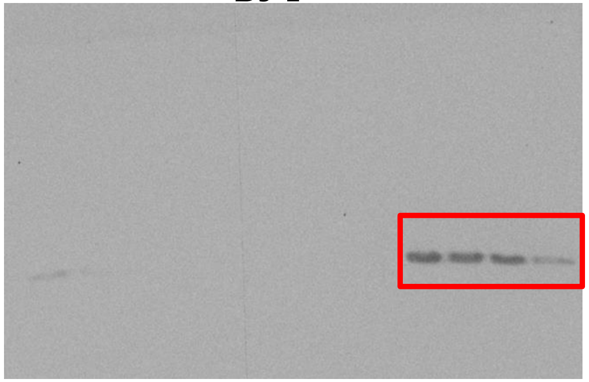

E163K

Tubulin

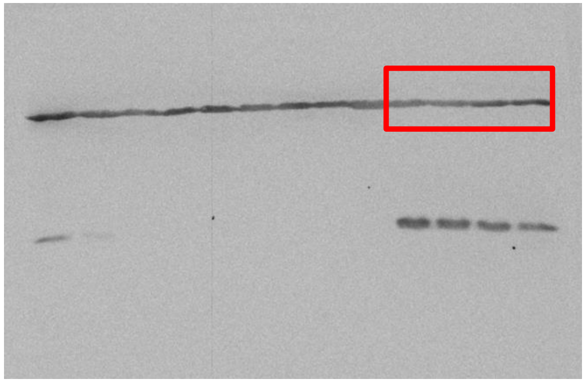

E163K

Figure 1

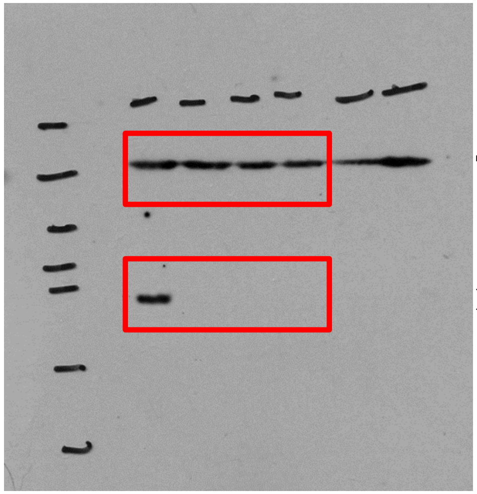

DJ-1

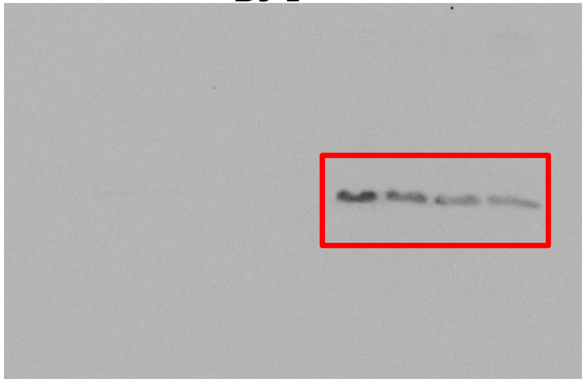

L172Q

Tubulin

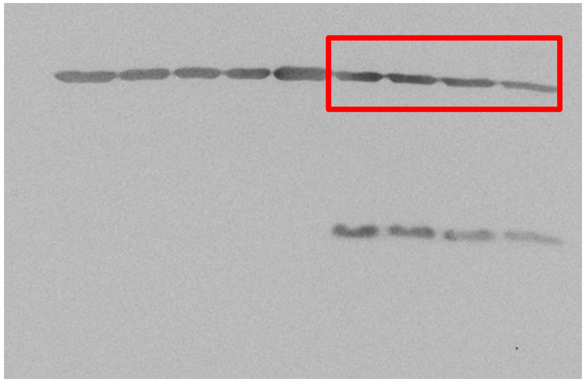

L172Q

Figure 1

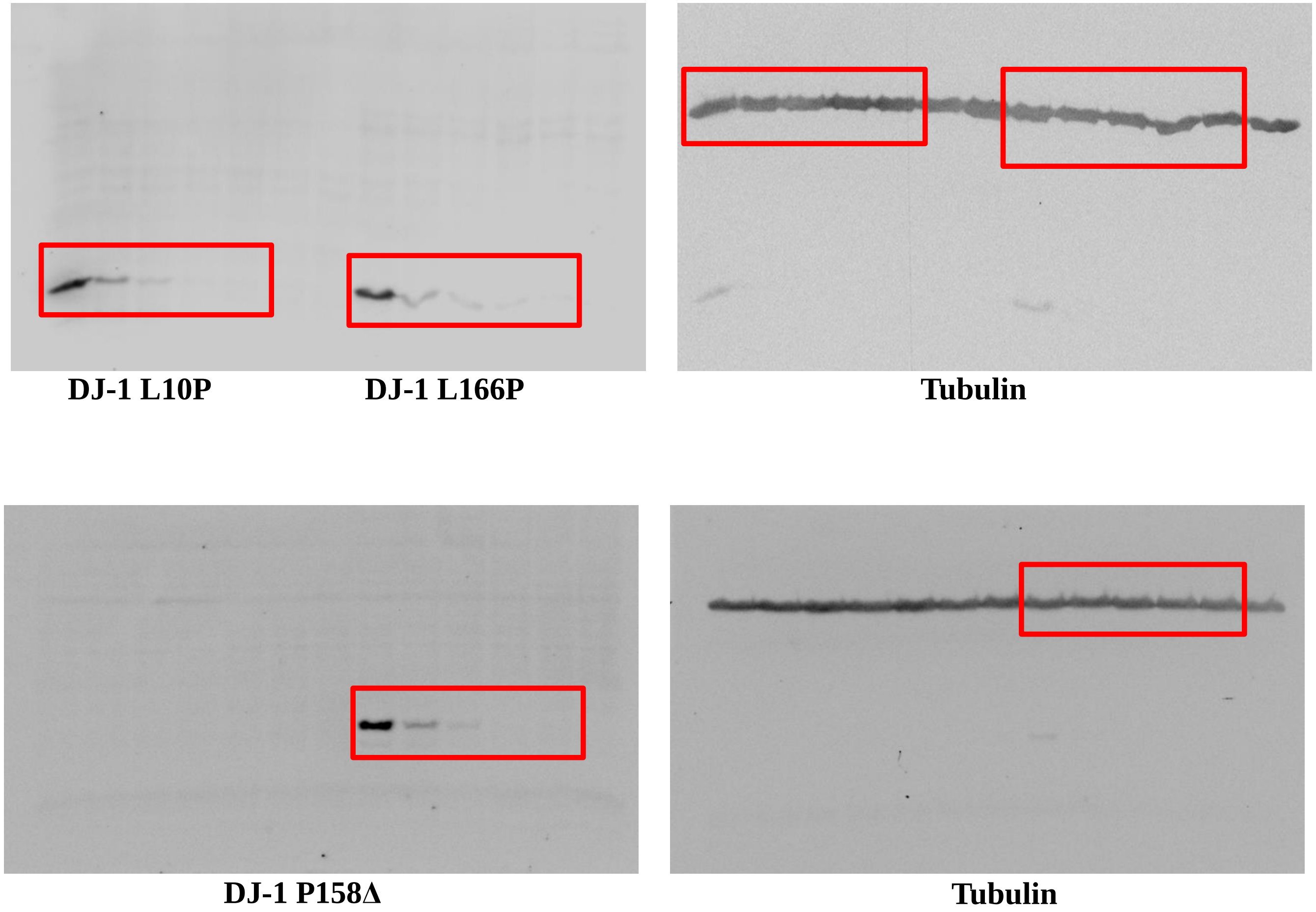

Figure 1

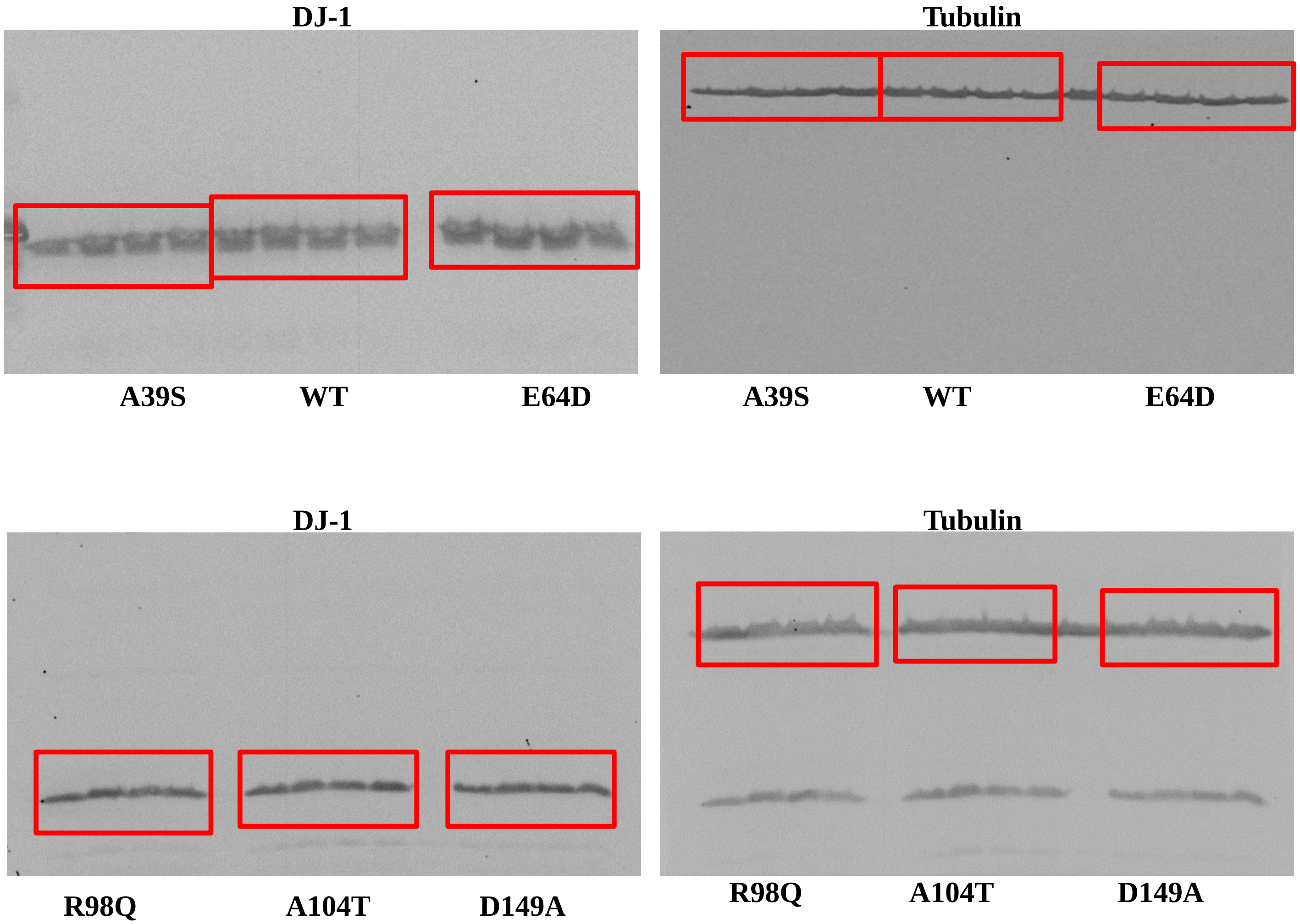
DJ-1
Tubulin
A39S
WT
E64D
A39S
WT
E64D
DJ-1
Tubulin
R98Q
A104T
D149A
R98Q
A104T
D149A

Figure 2

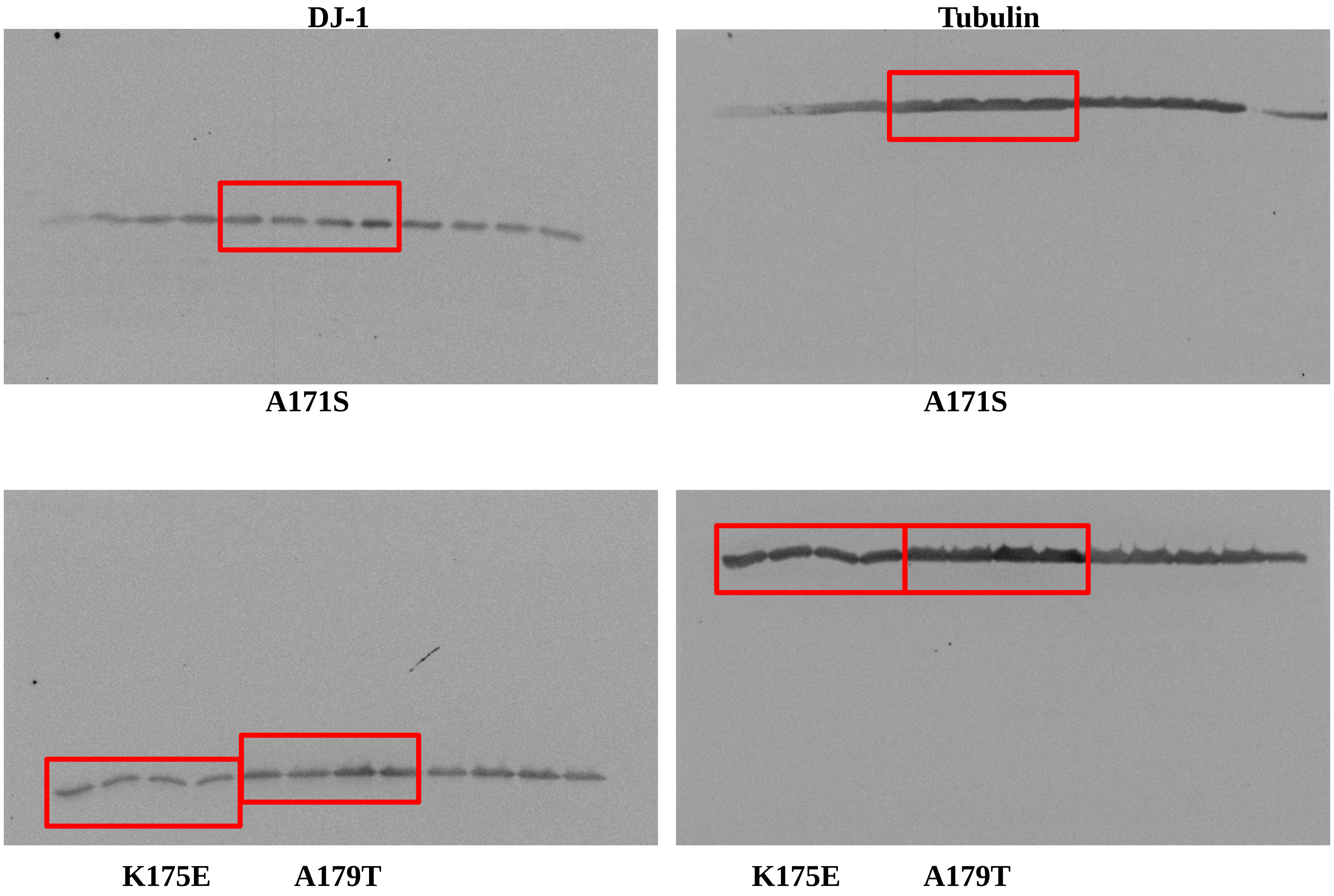

Figure 2

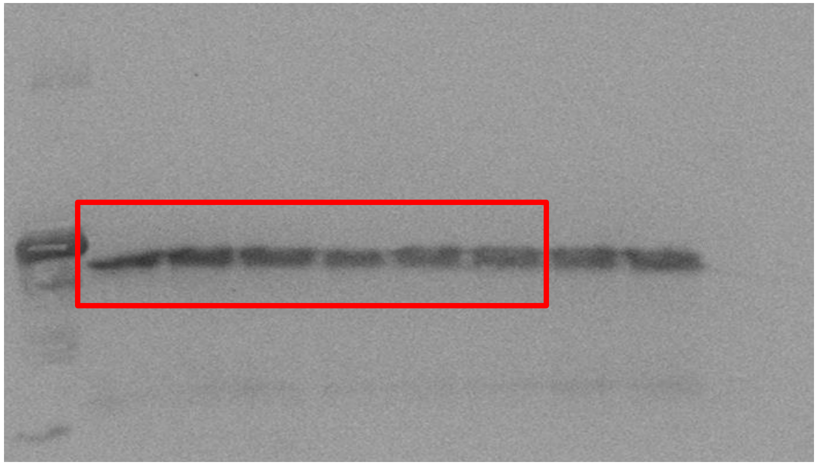

DJ-1 WT

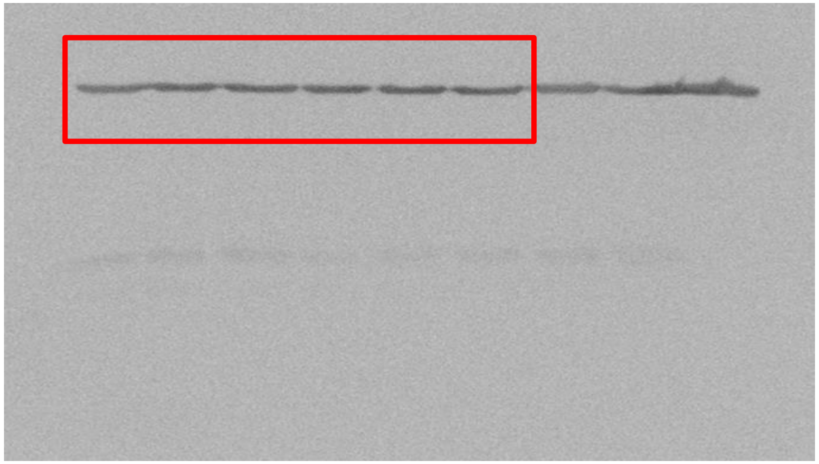

Tubulin

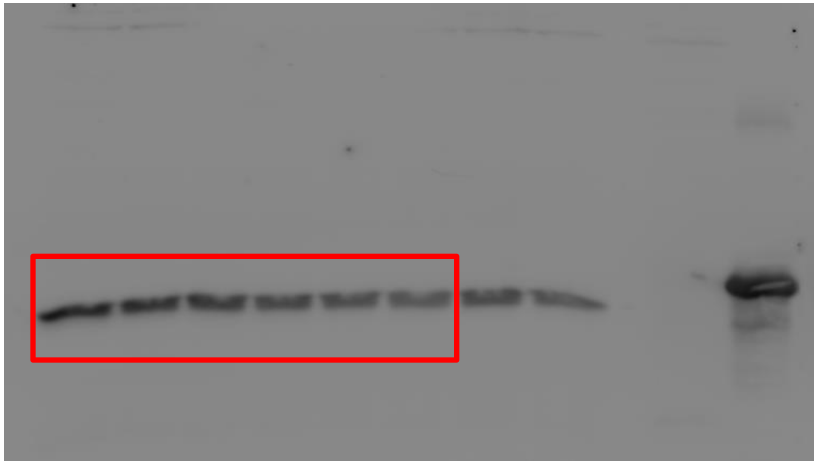

DJ-1 M26I

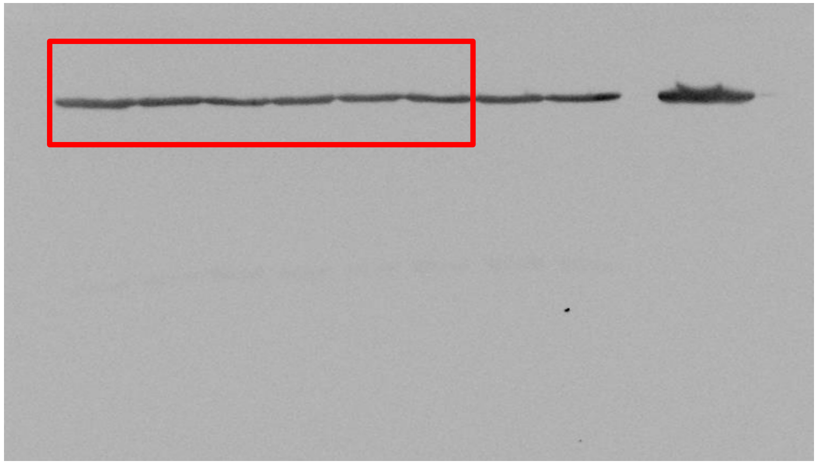

Tubulin

Figure 3

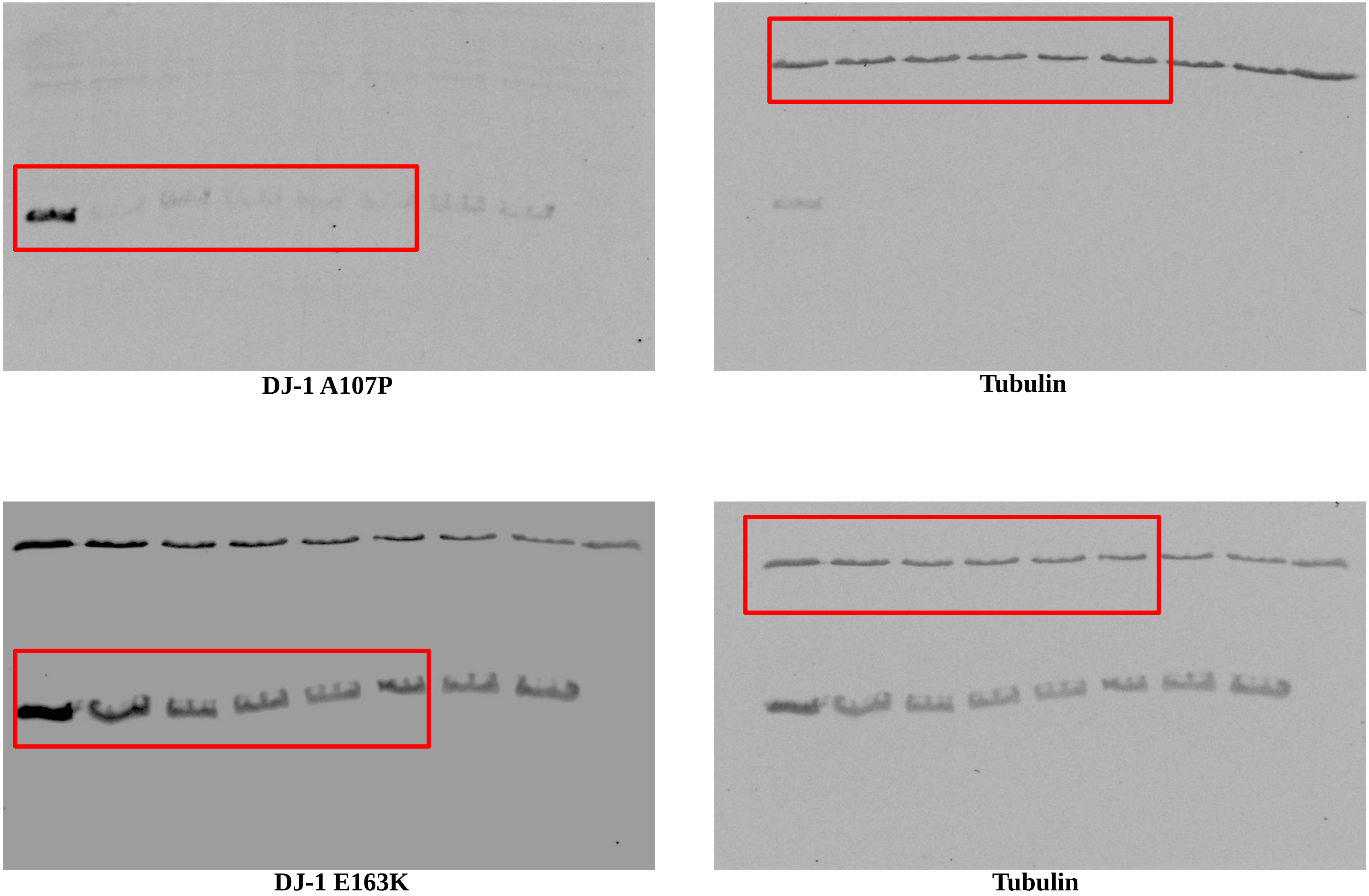

Figure 3

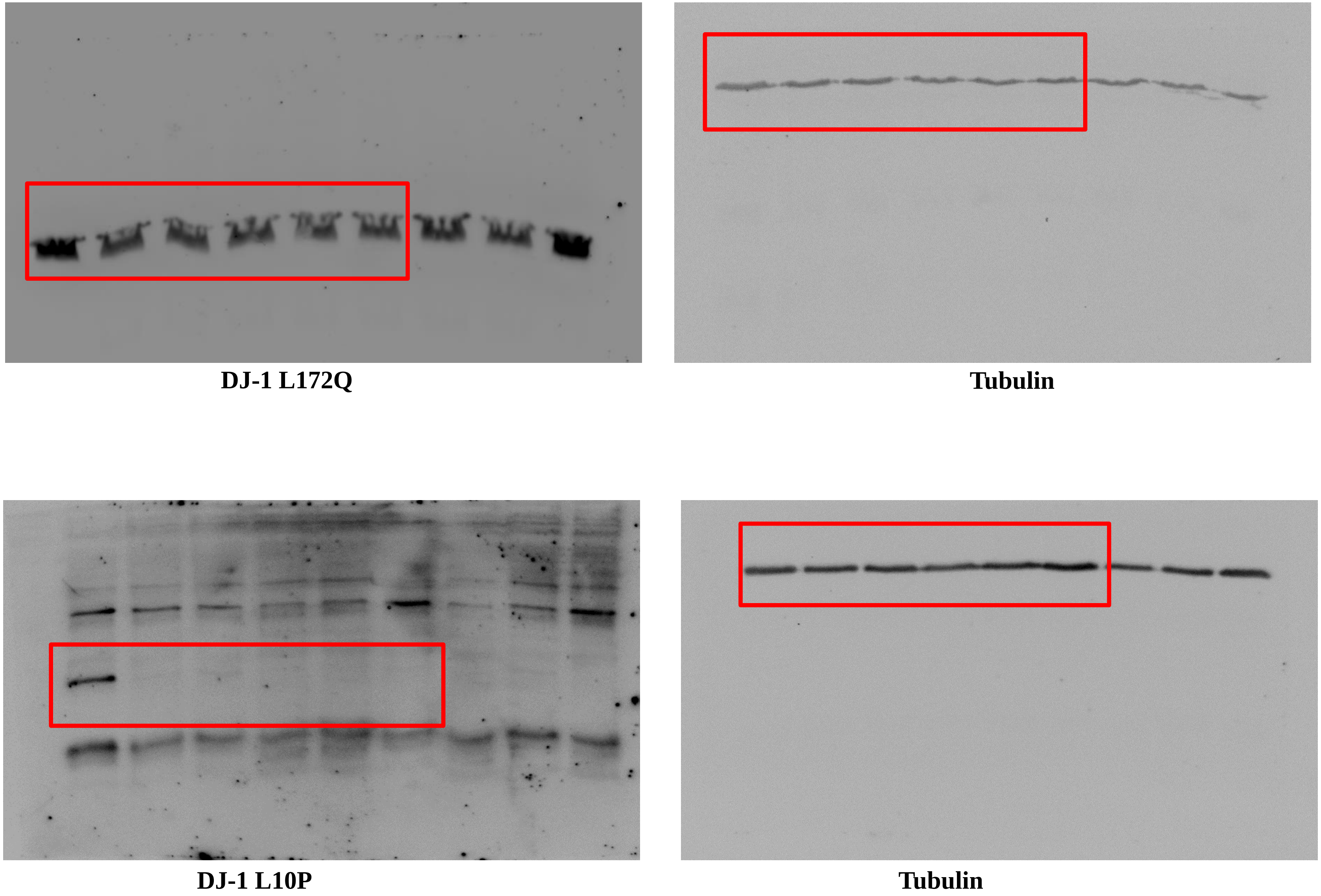

Figure 3

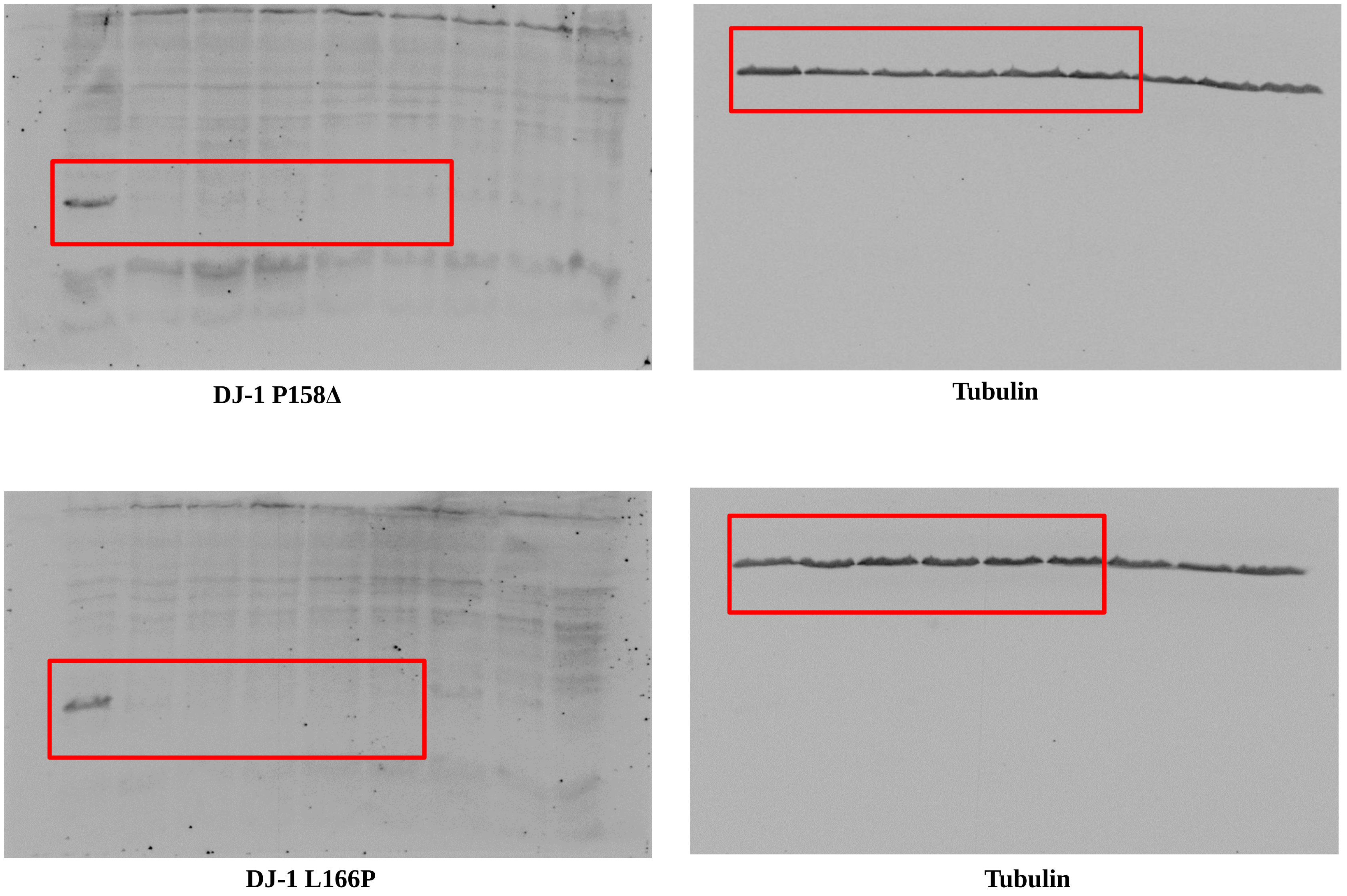

Figure 3

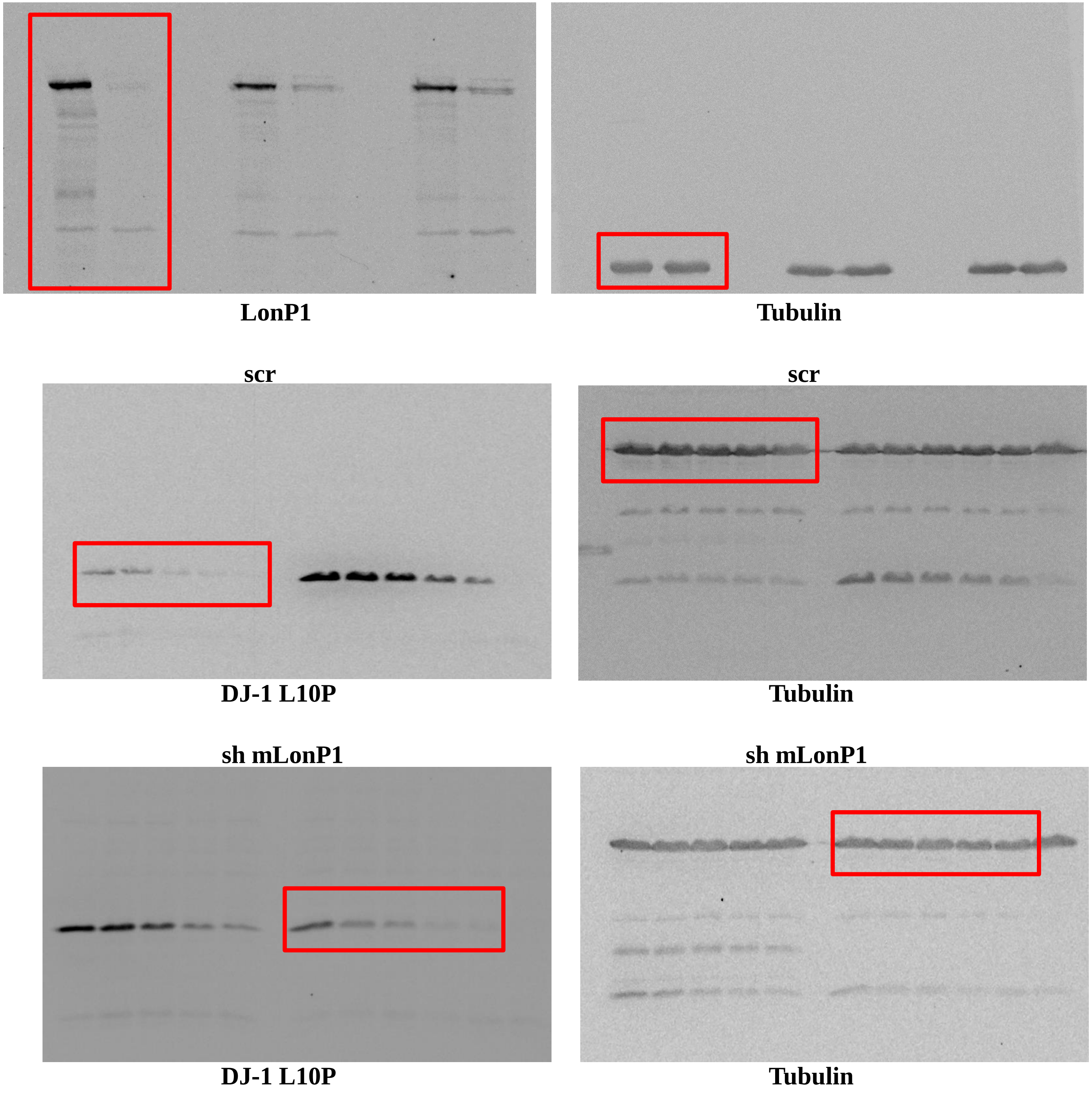
LonP1
Tubulin
scr
scr
DJ-1 L10P
Tubulin
sh mLonP1
sh mLonP1
DJ-1 L10P
Tubulin

Figure 5

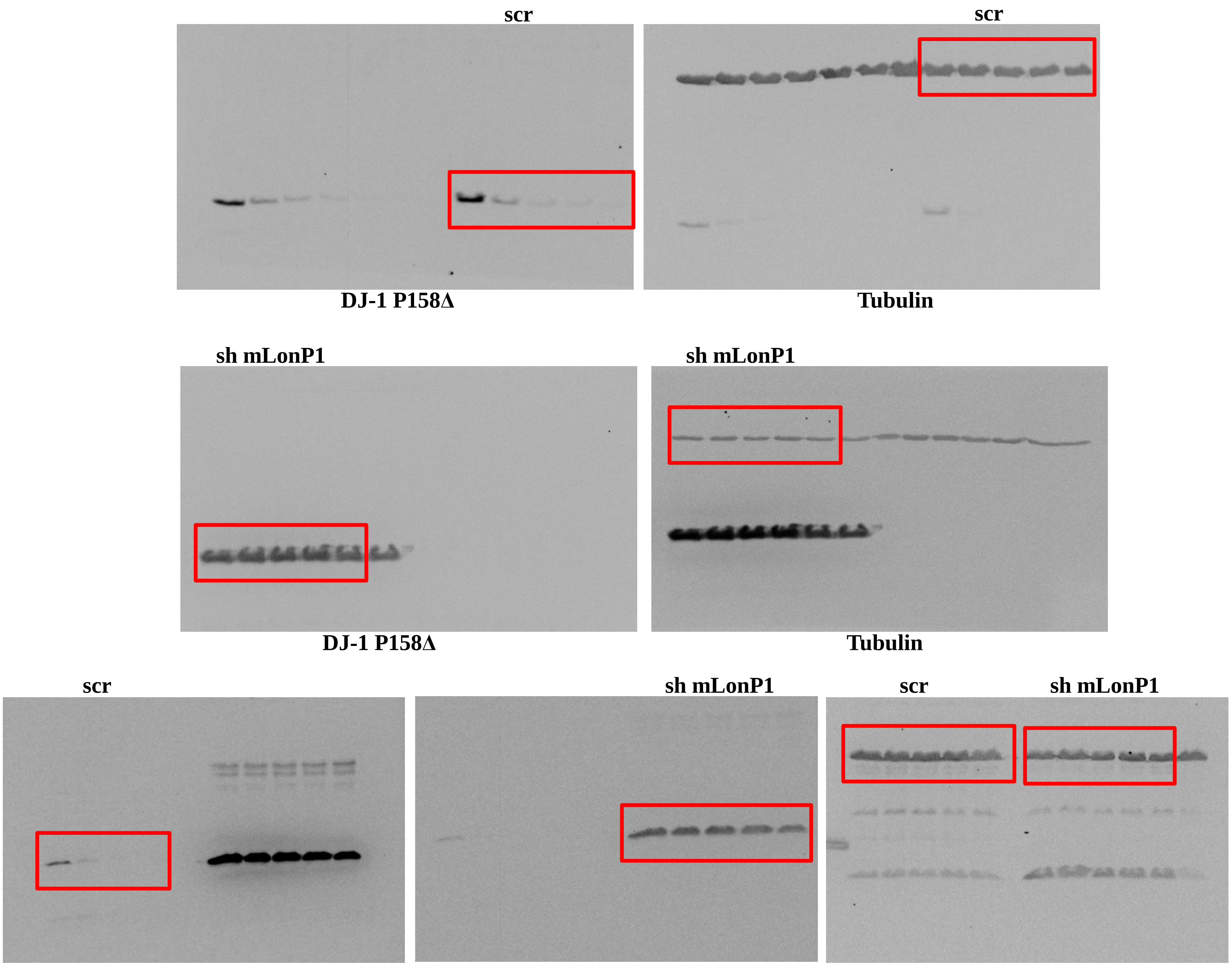
scr
scr
DJ-1 P158Δ
Tubulin
sh mLonP1
sh mLonP1
DJ-1 P158Δ
Tubulin
scr
sh mLonP1
scr
sh mLonP1
DJ-1 L166P
DJ-1 L166P
Tubulin

Figure 5

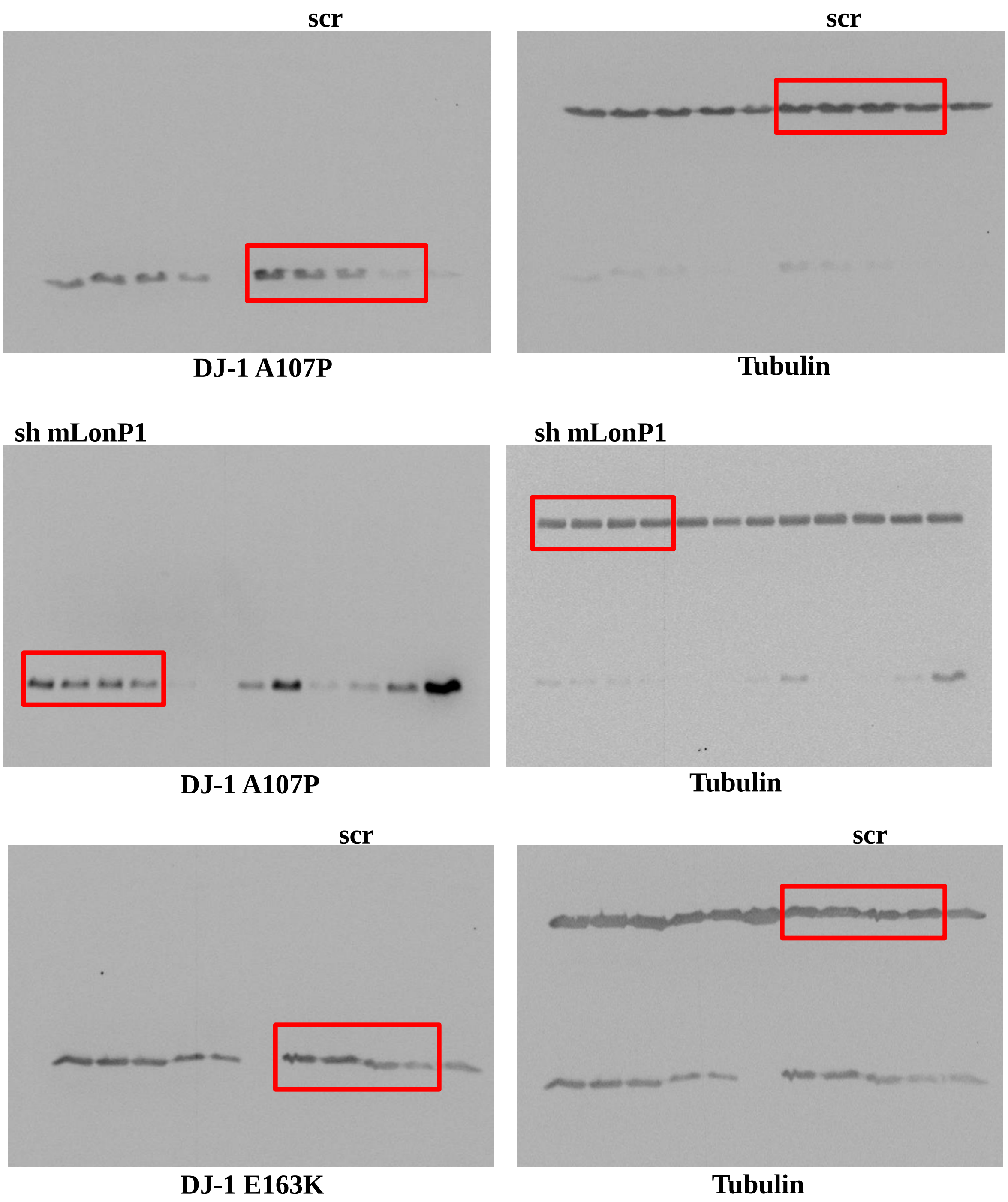
scr
DJ-1 A107P
scr
Tubulin
sh mLonP1
DJ-1 A107P
sh mLonP1
Tubulin
scr
DJ-1 E163K
scr
Tubulin

Figure 5

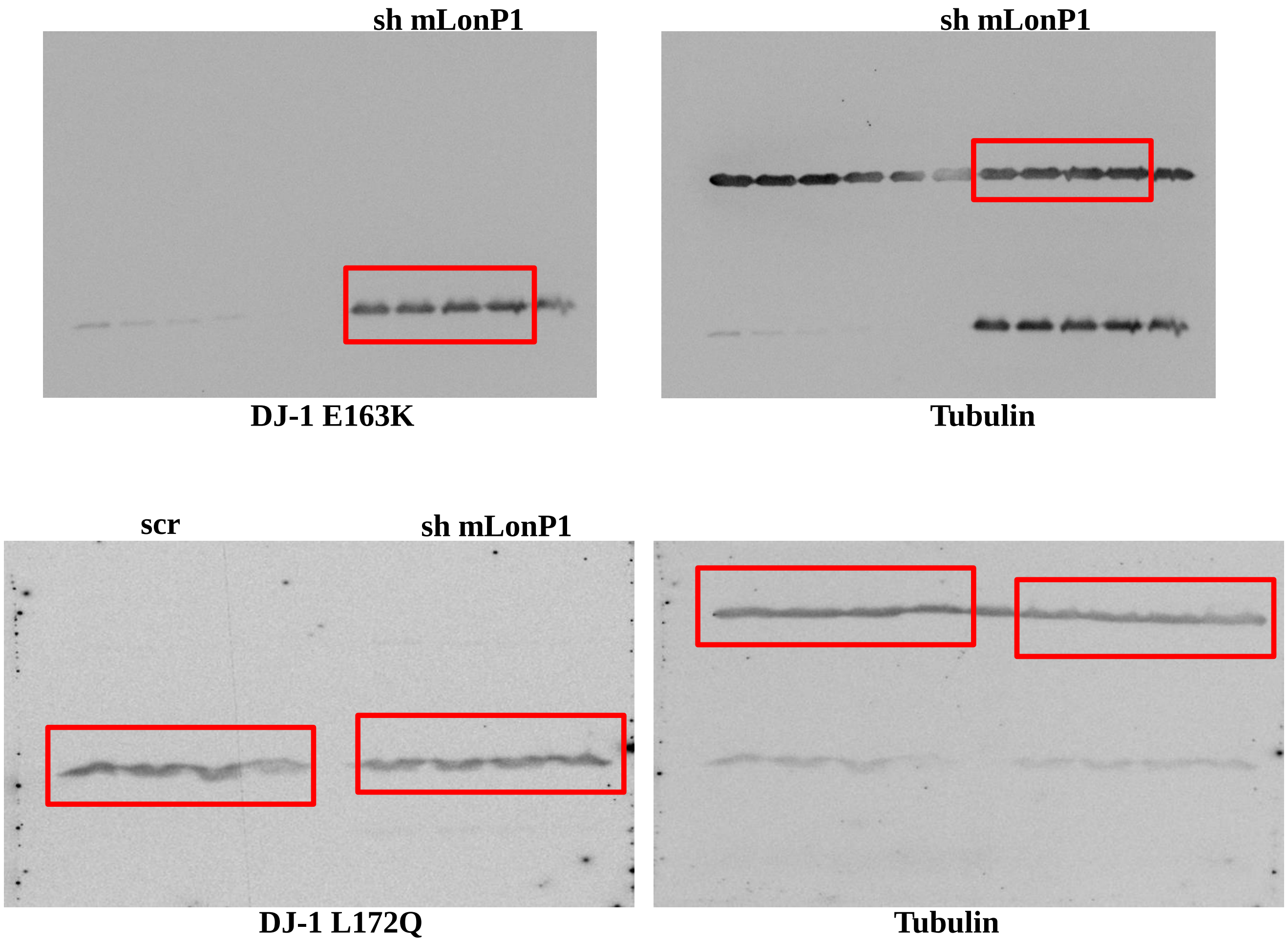
sh mLonP1
DJ-1 E163K
sh mLonP1
Tubulin
scr
sh mLonP1
DJ-1 L172Q
Tubulin

Figure 5

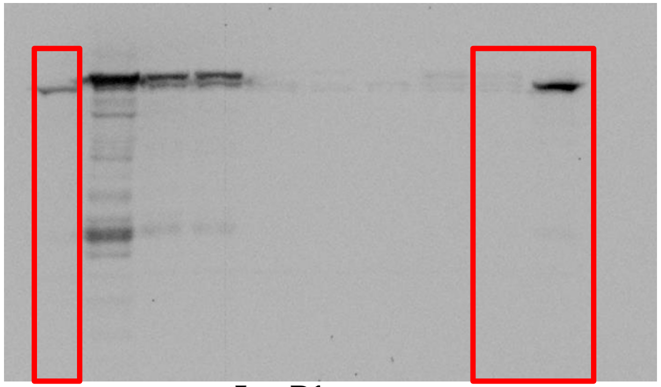

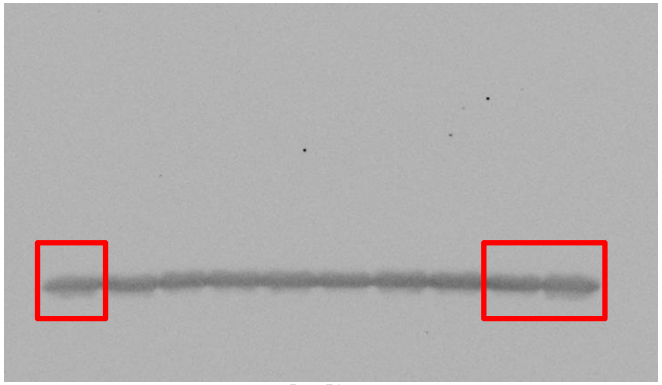

Figure 6

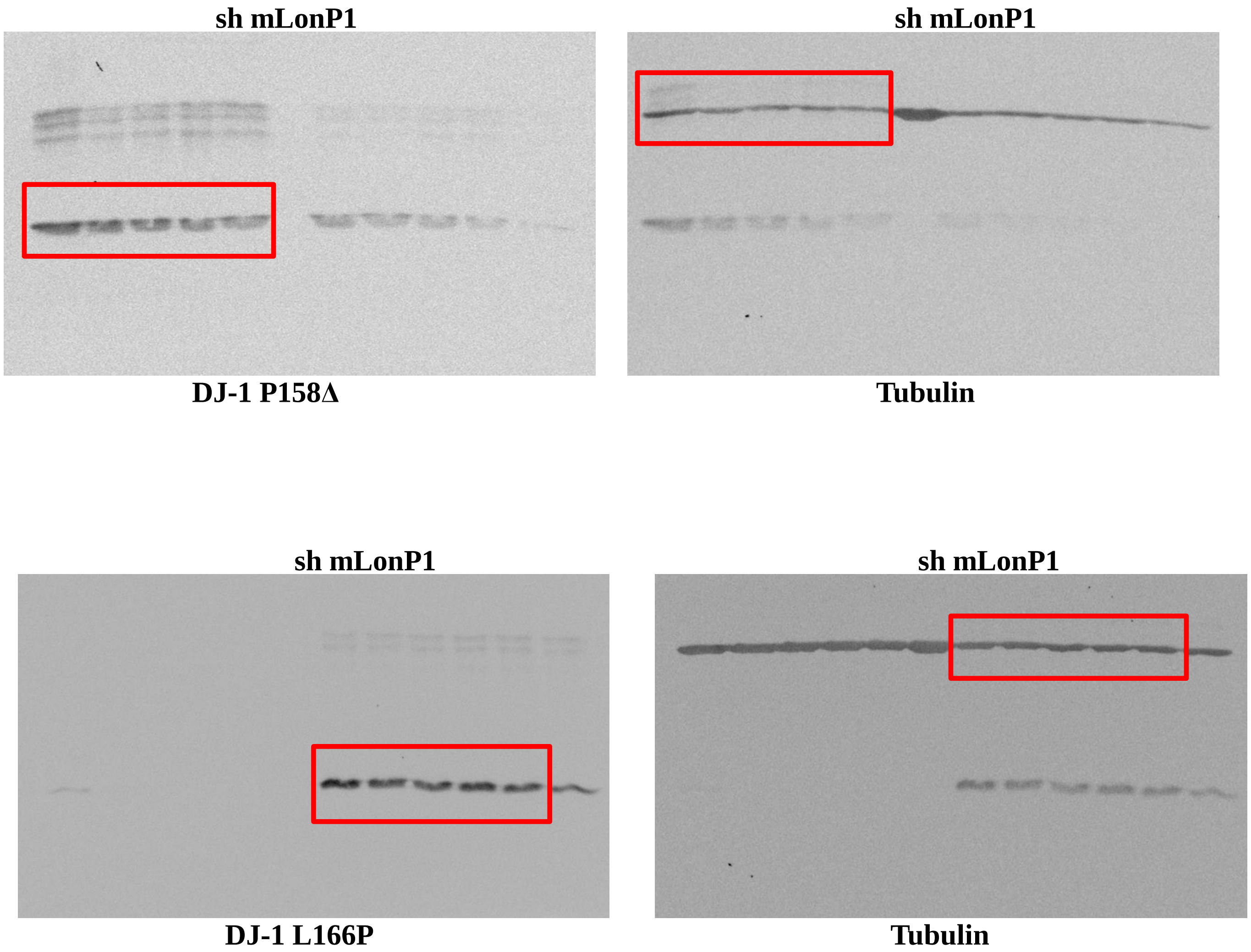
sh mLonP1
sh mLonP1
DJ-1 P158Δ
Tubulin
sh mLonP1
sh mLonP1
DJ-1 L166P
Tubulin

Figure 6

Title: Mitochondrial LonP1 protease is implicated in the degradation of unstable Parkinson disease-associated *DJ-1/PARK 7* missense mutants.

Authors: Raúl Sánchez-Lanzas and José G. Castaño

Uncropped full-length blots corresponding to the immunoblots used in the figures of the paper.

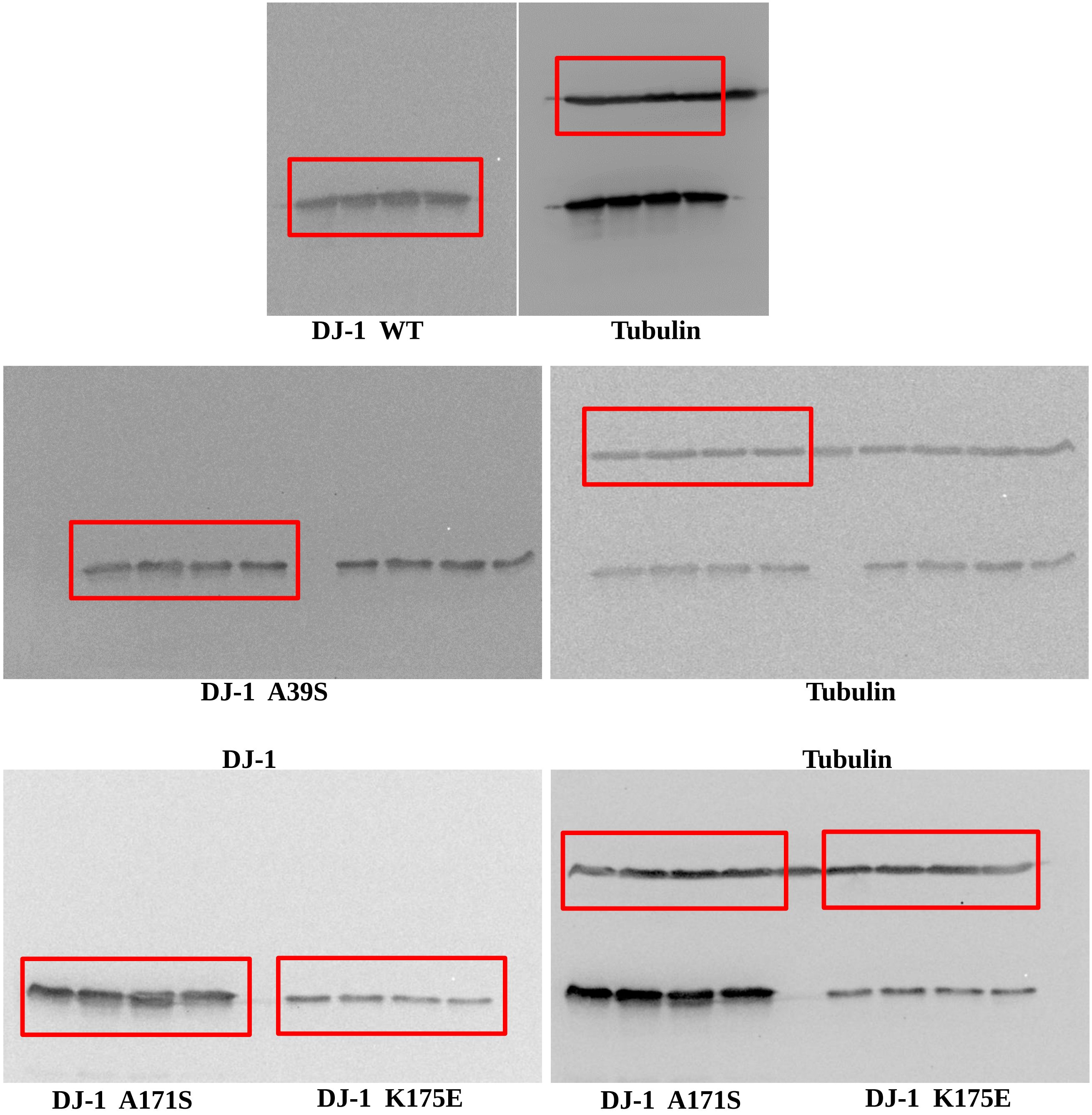

Supplementary Figure 1

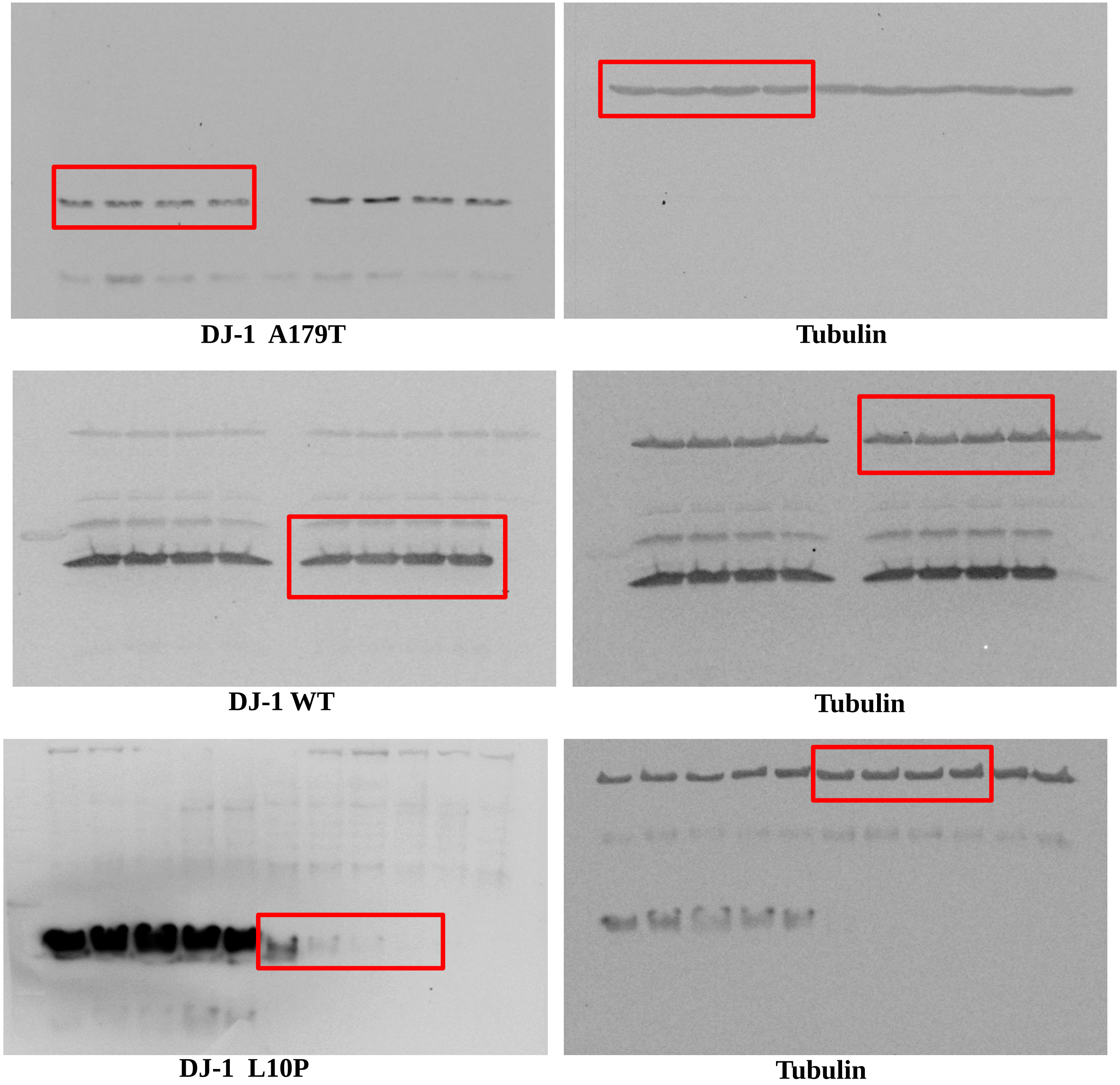

Supplementary Figure 1

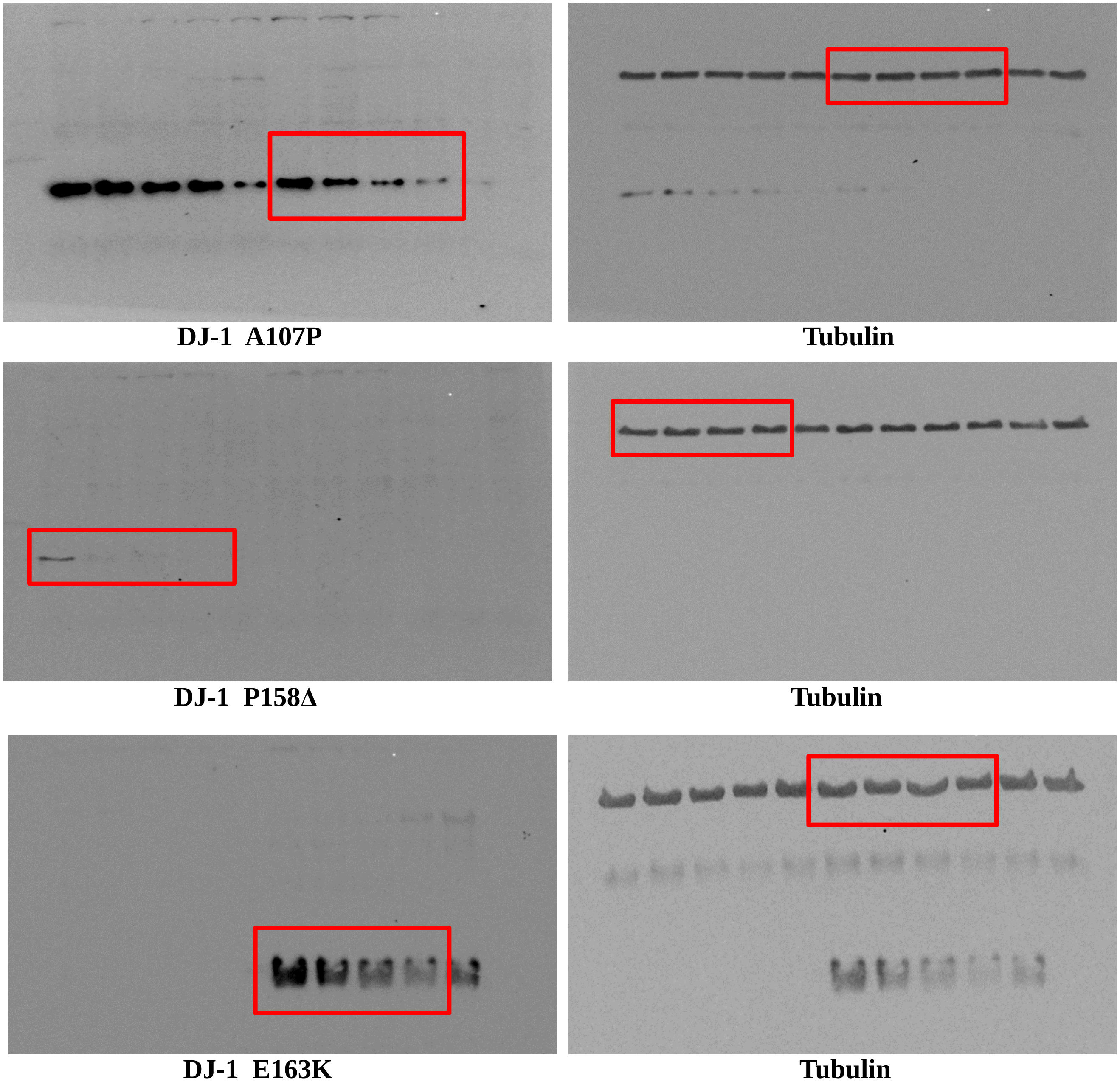

Supplementary Figure 1

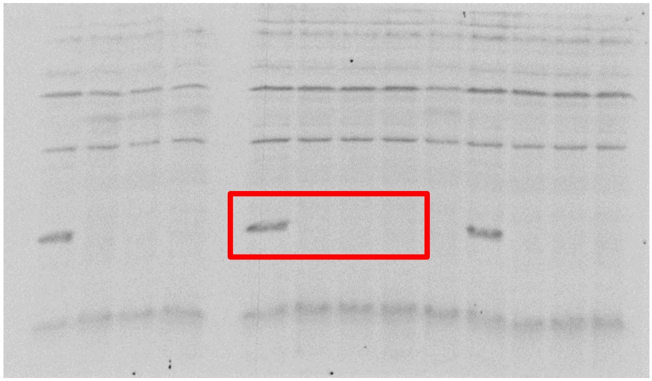

DJ-1 L166P

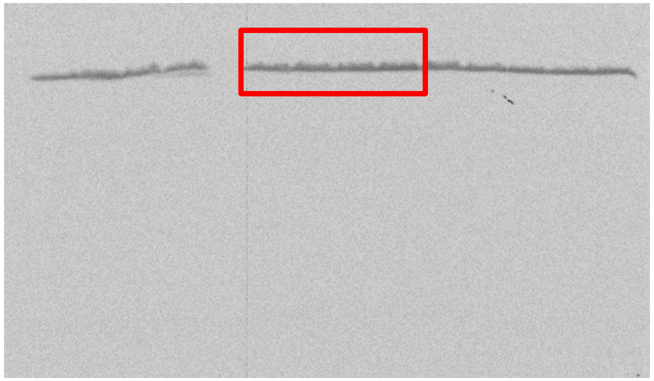

Tubulin

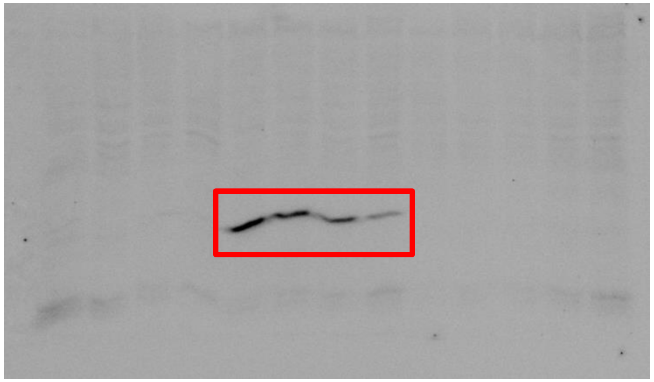

DJ-1 L172Q

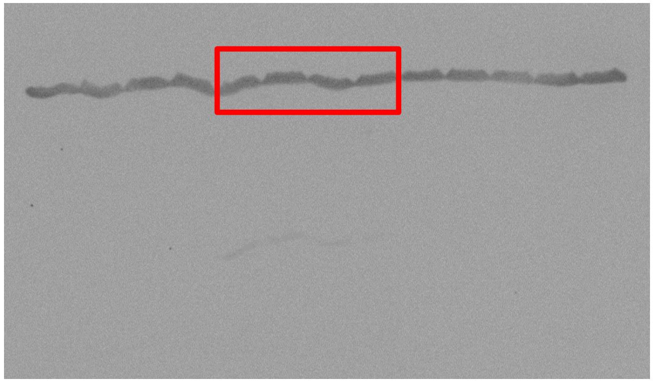

Tubulin

Supplementary Figure 1

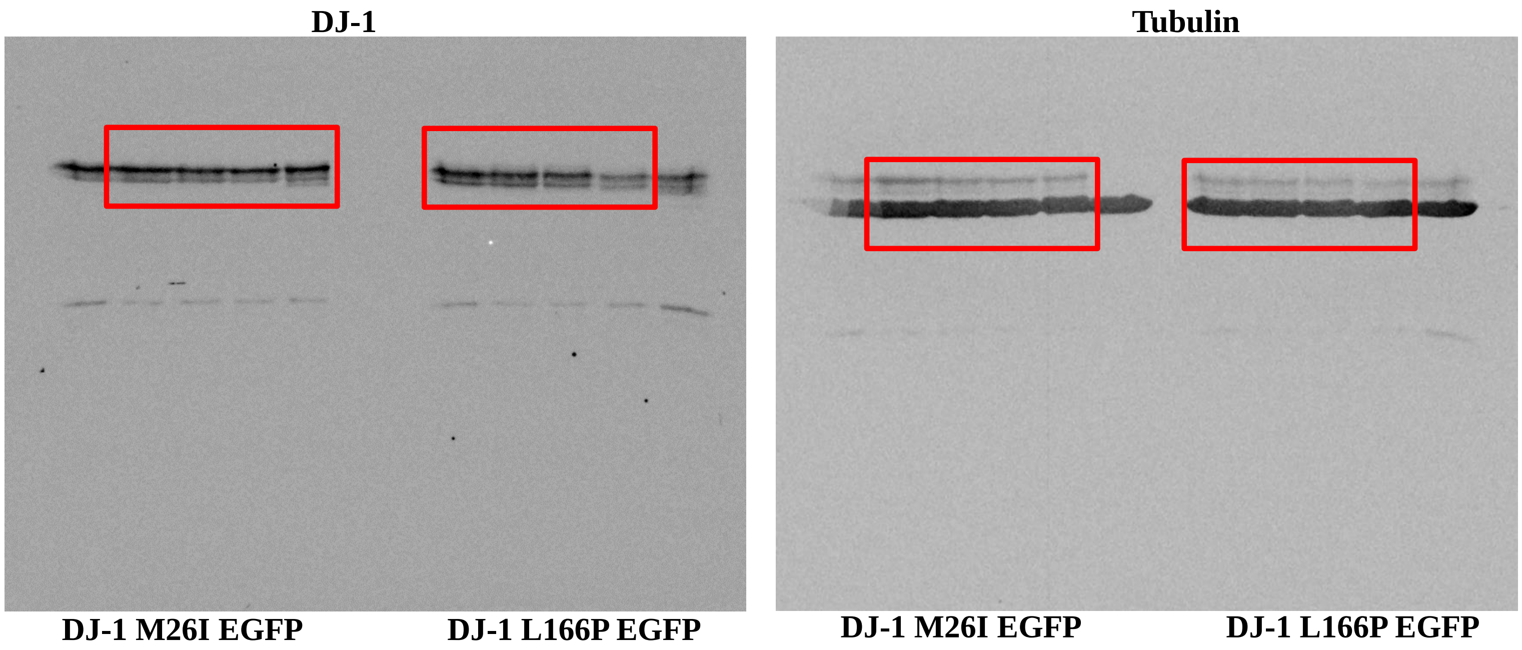

Supplementary Figure 3

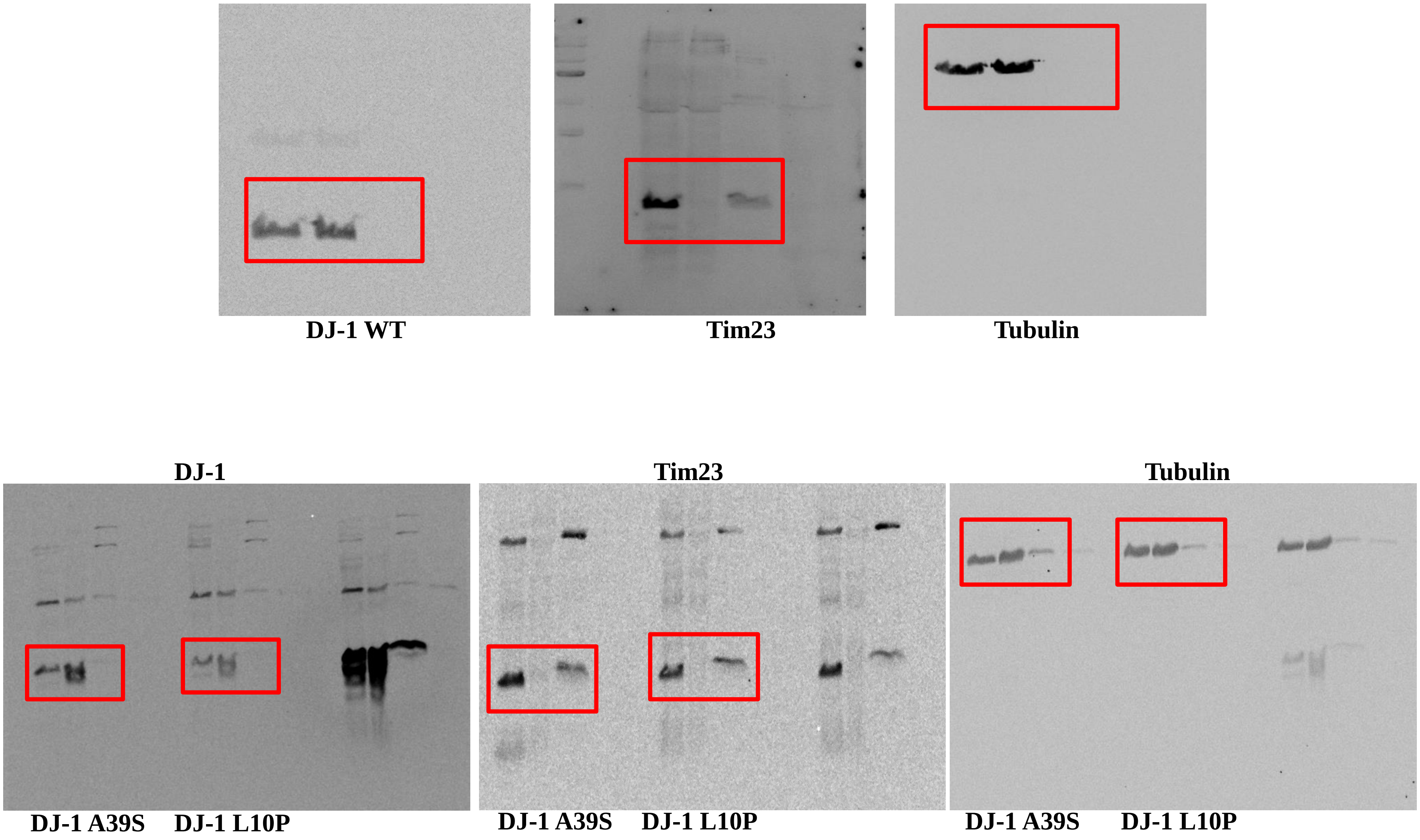

Supplementary Figure 4

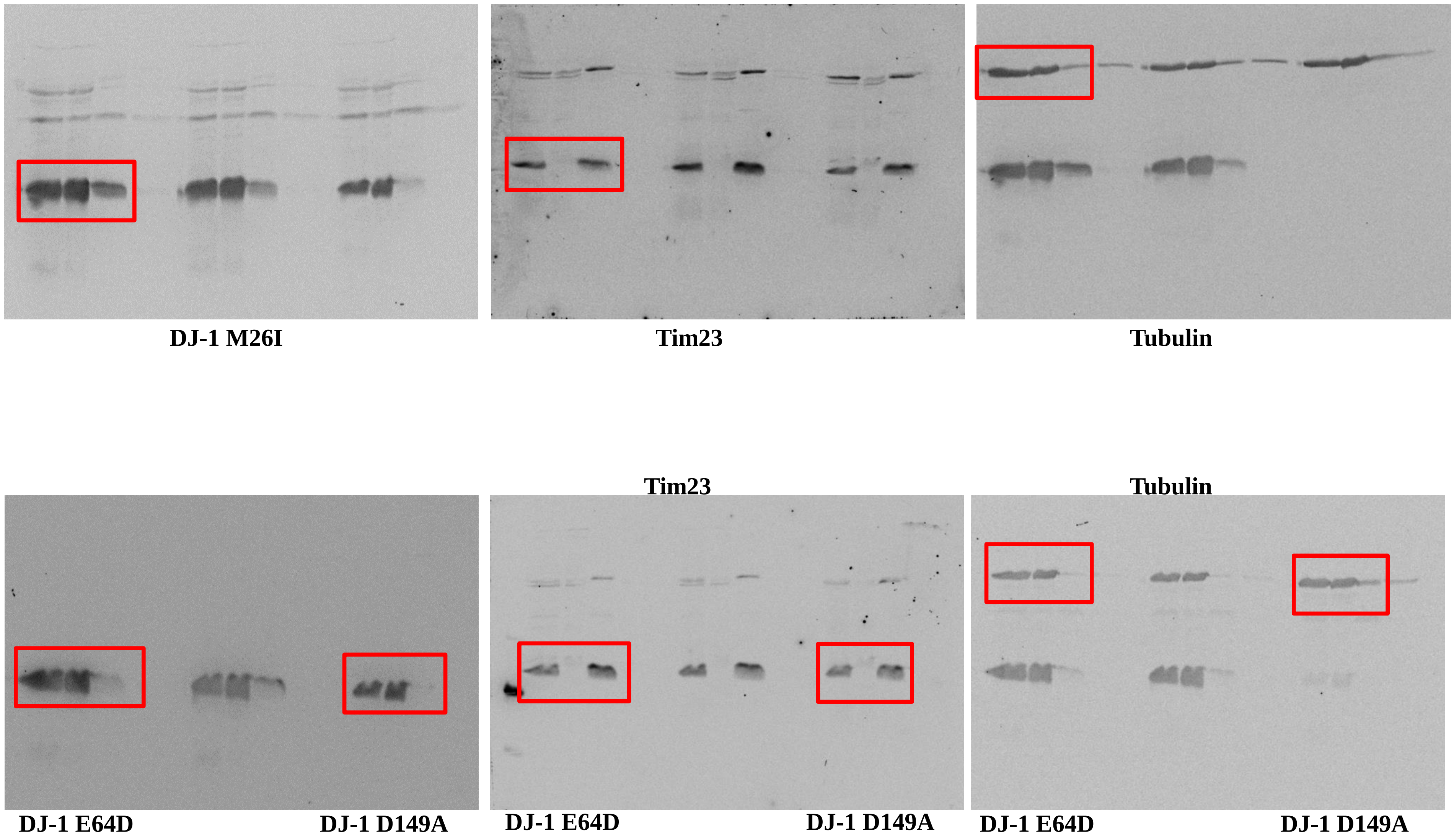

Supplementary Figure 4

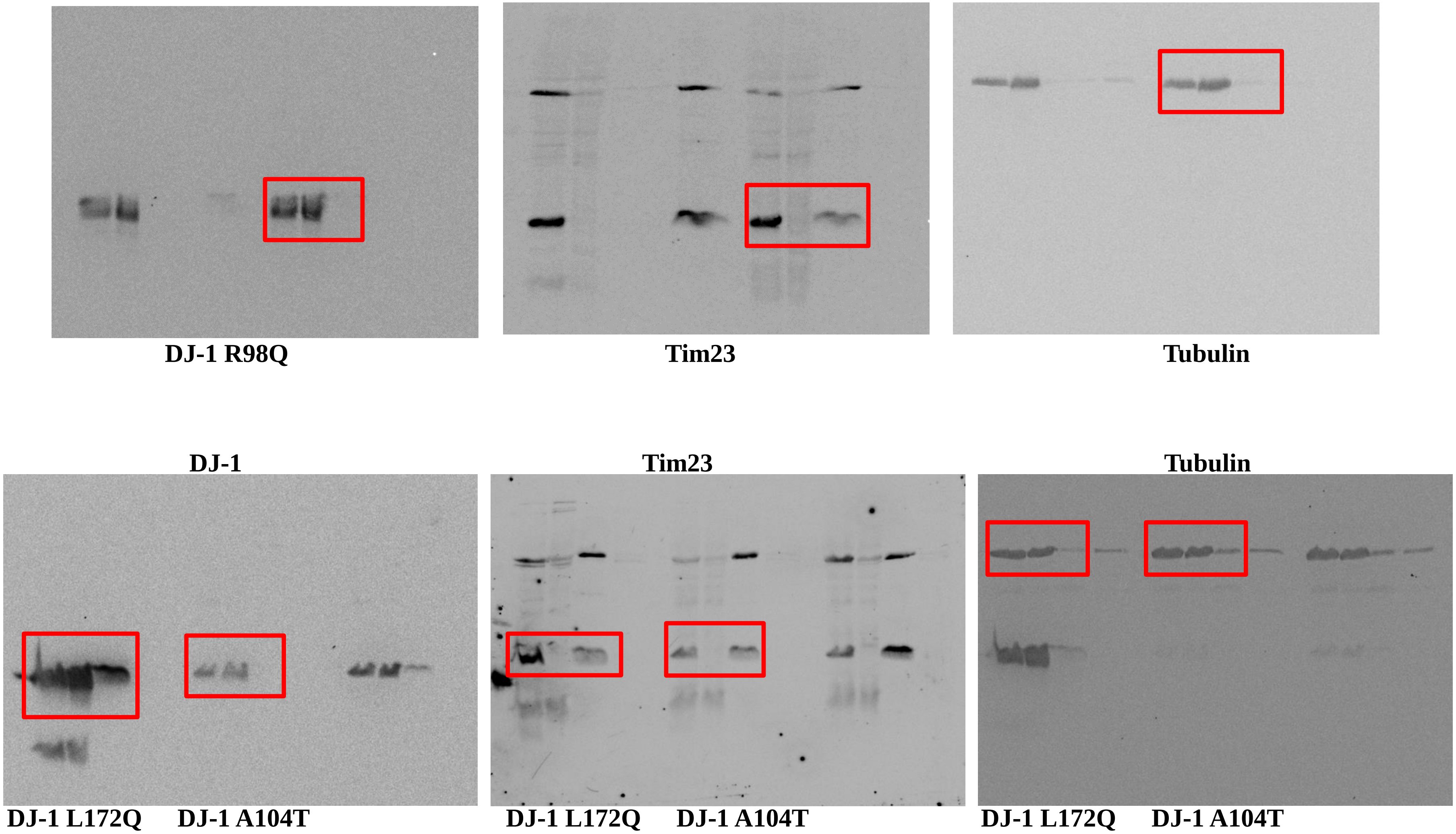

Supplementary Figure 4

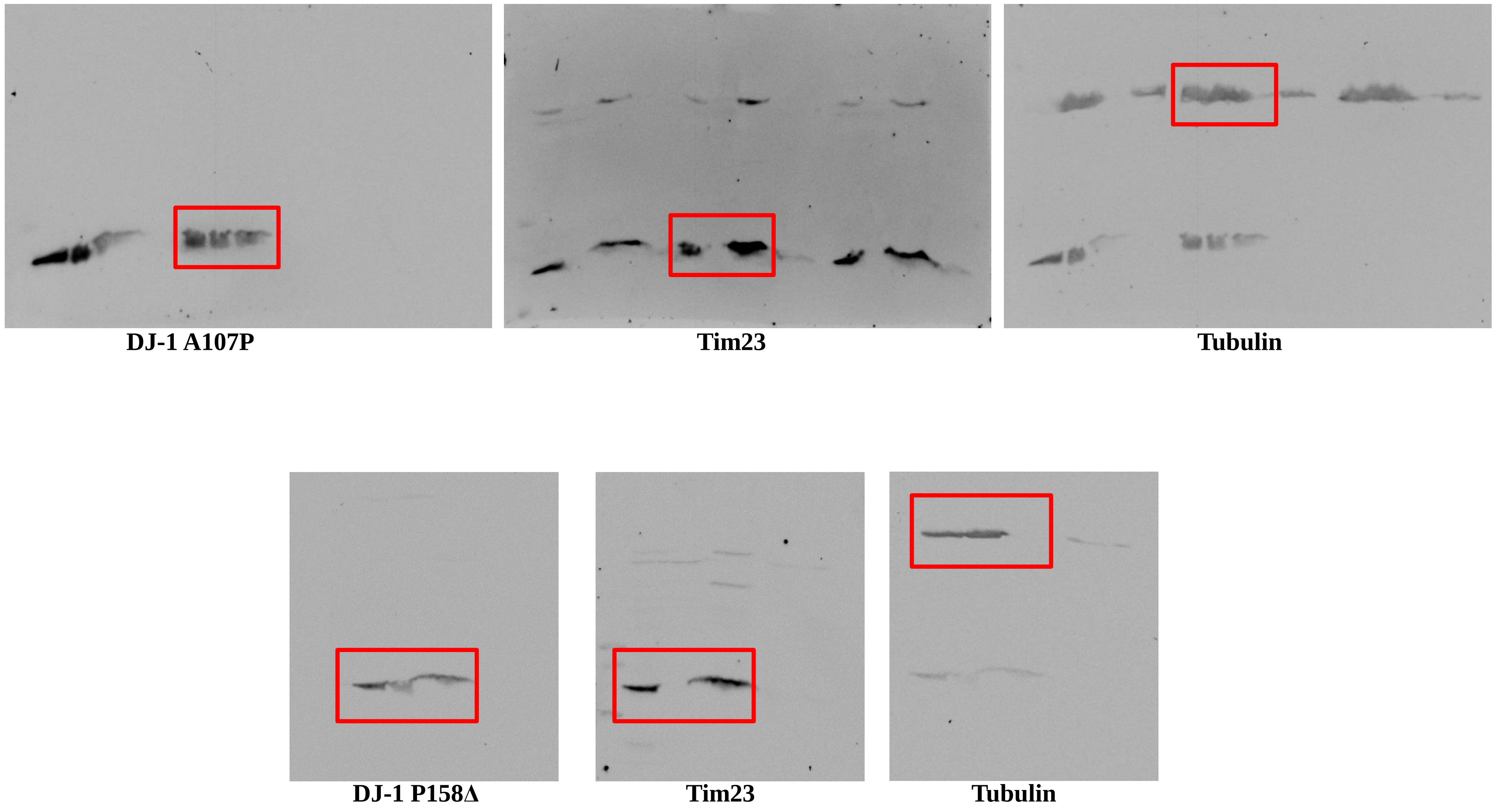

Supplementary Figure 4

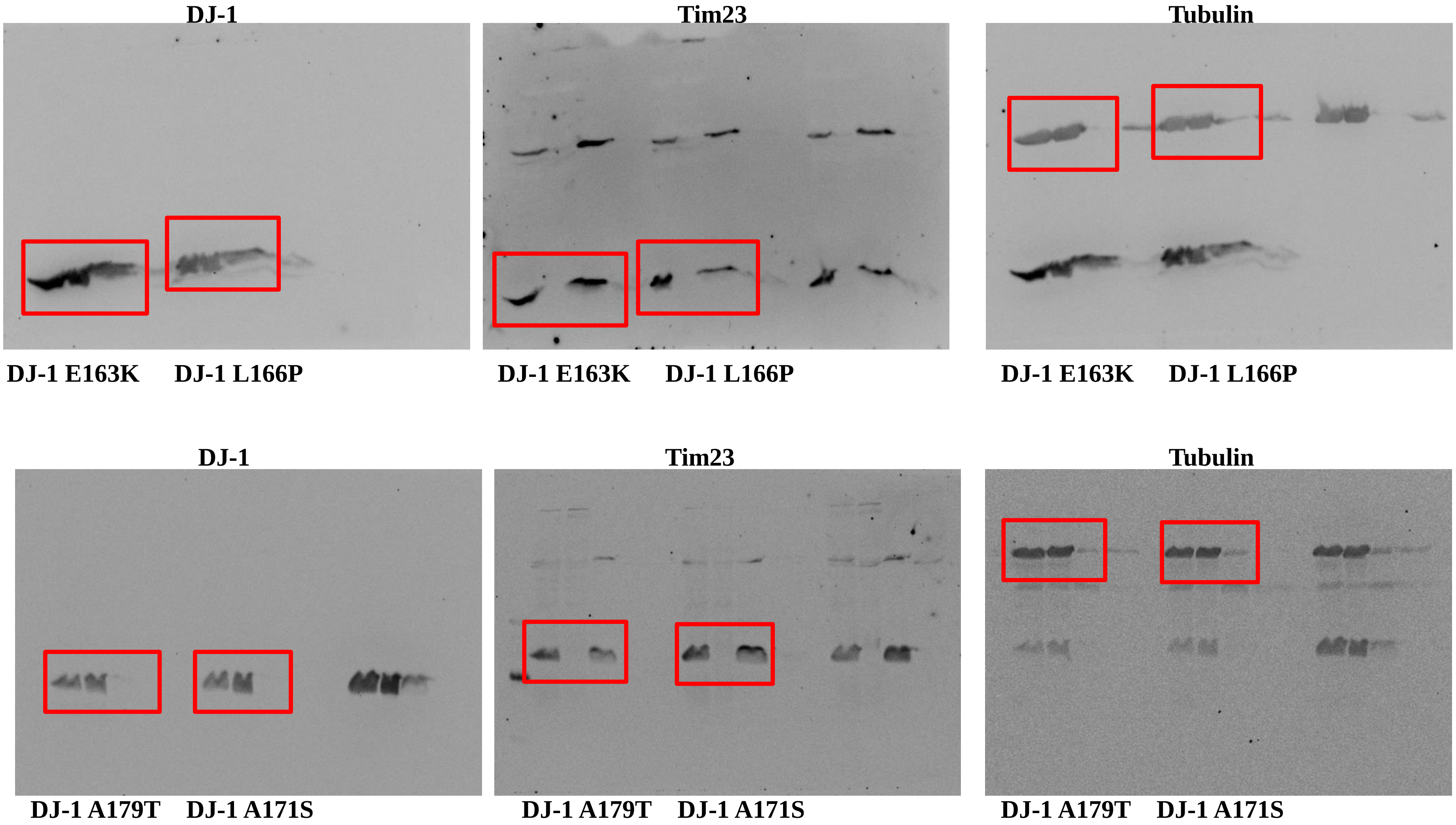

Supplementary Figure 4

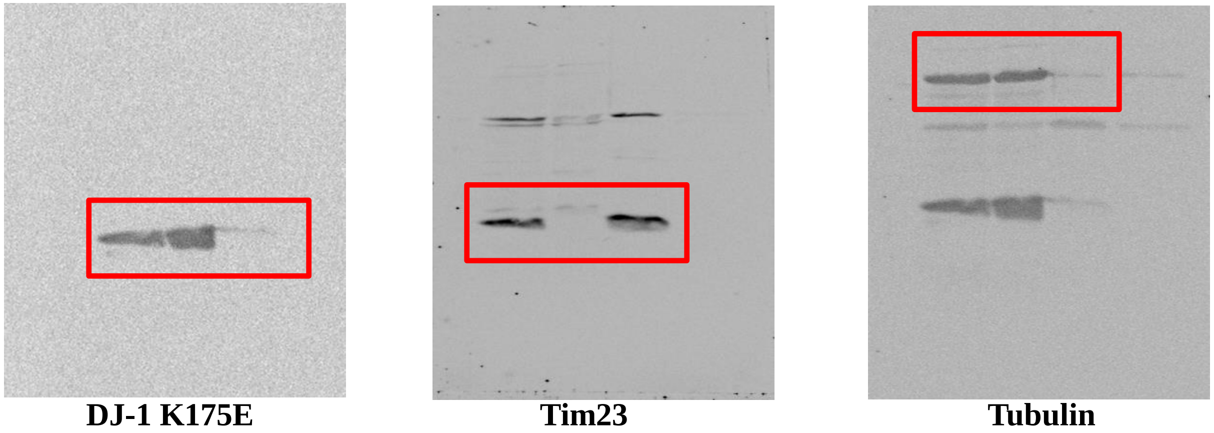

Supplementary Figure 4

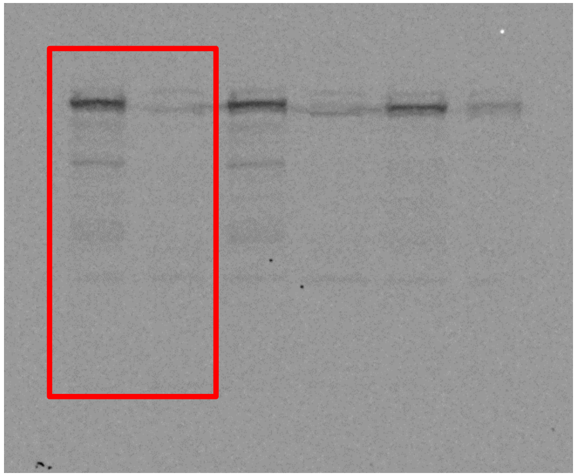

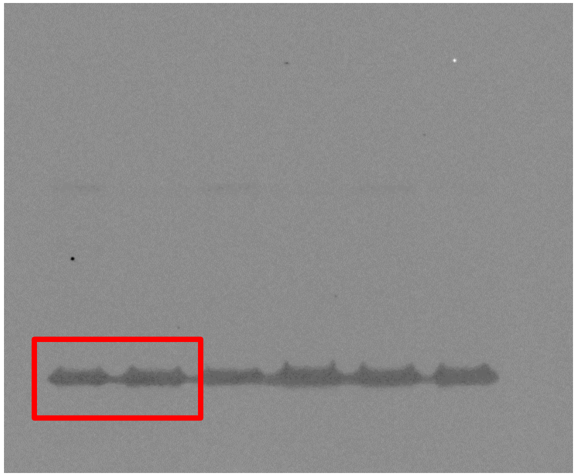

LonP1 Tubulin

Supplementary Figure 5

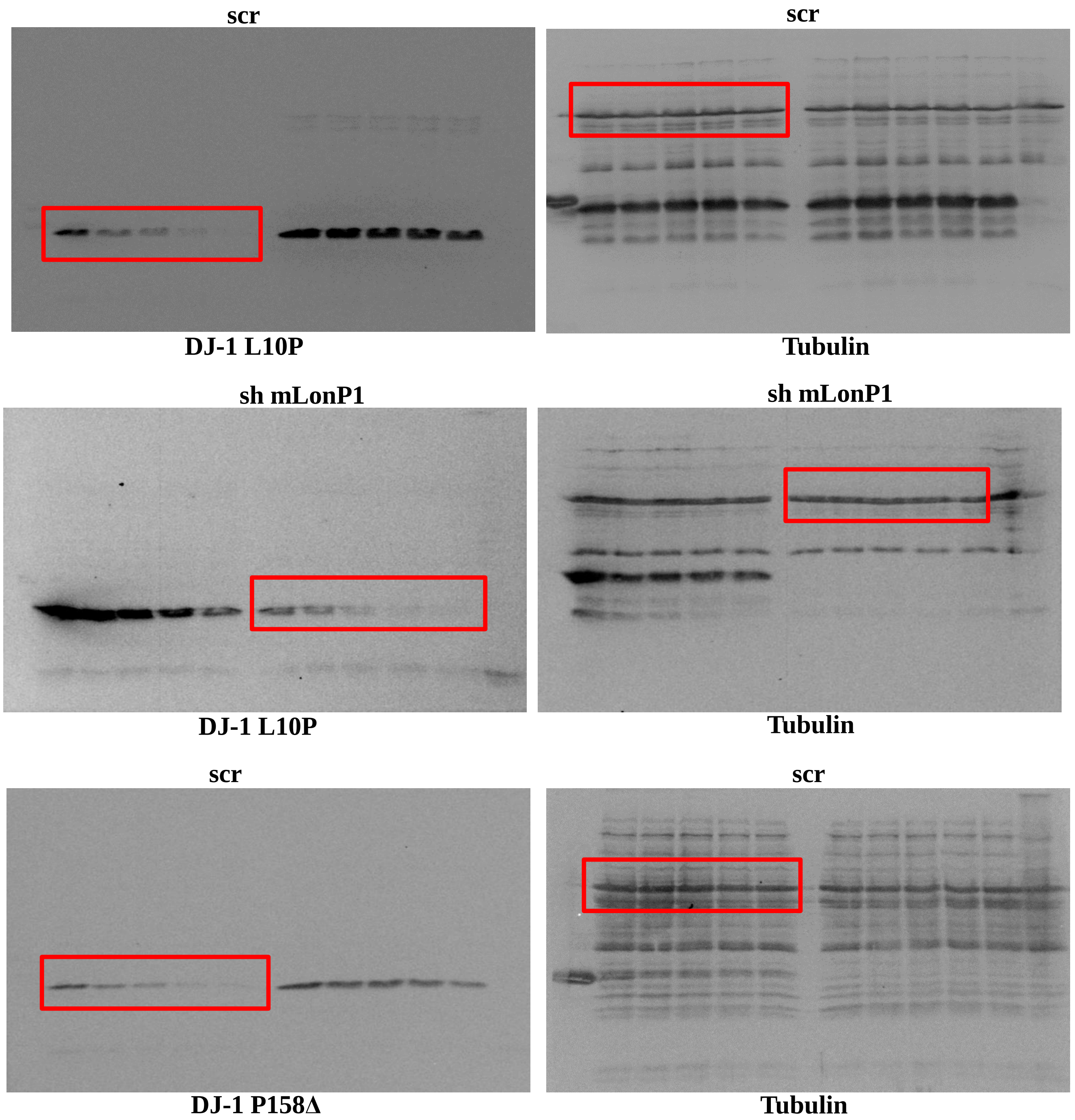

Supplementary Figure 5

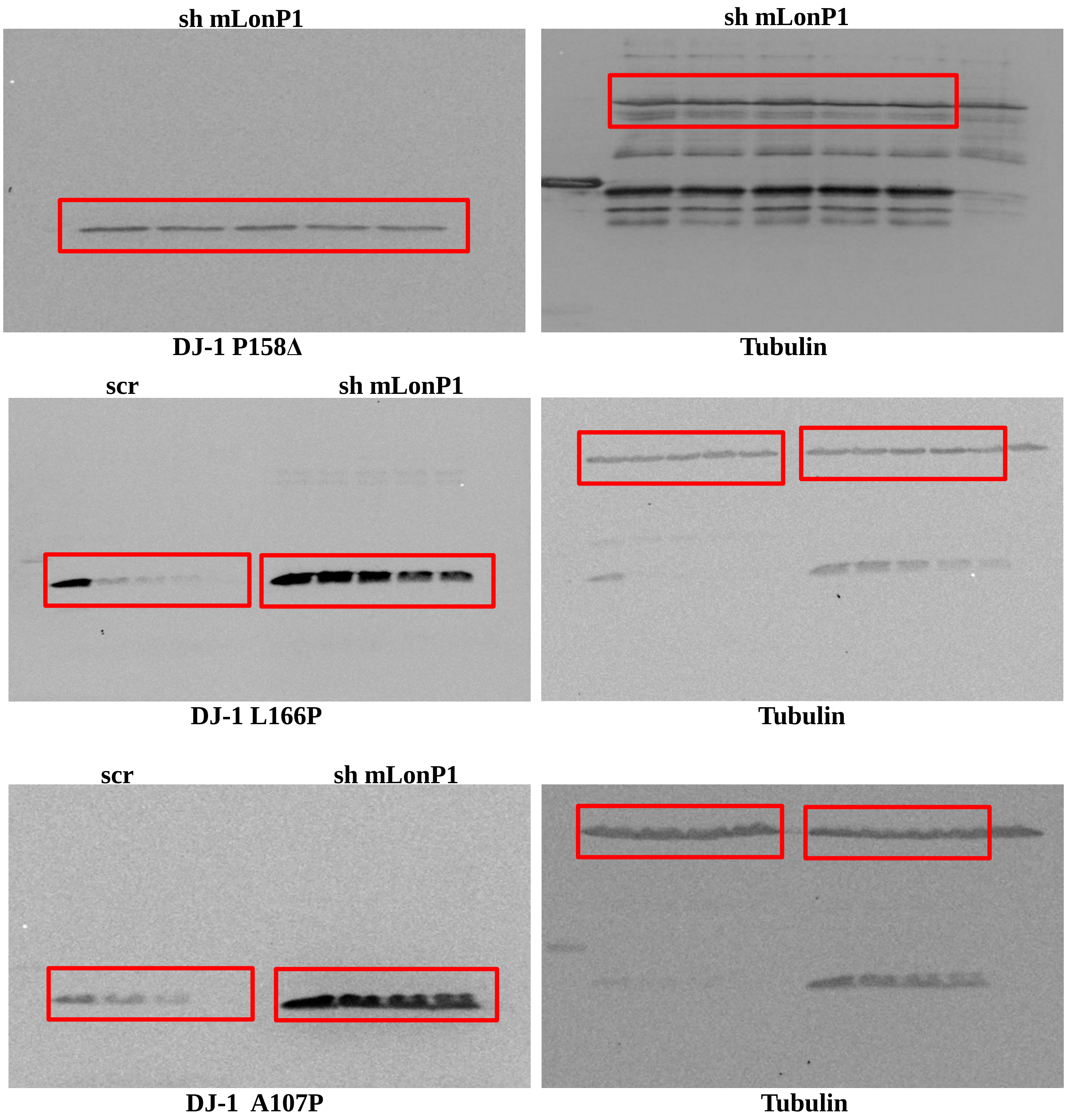

Supplementary Figure 5

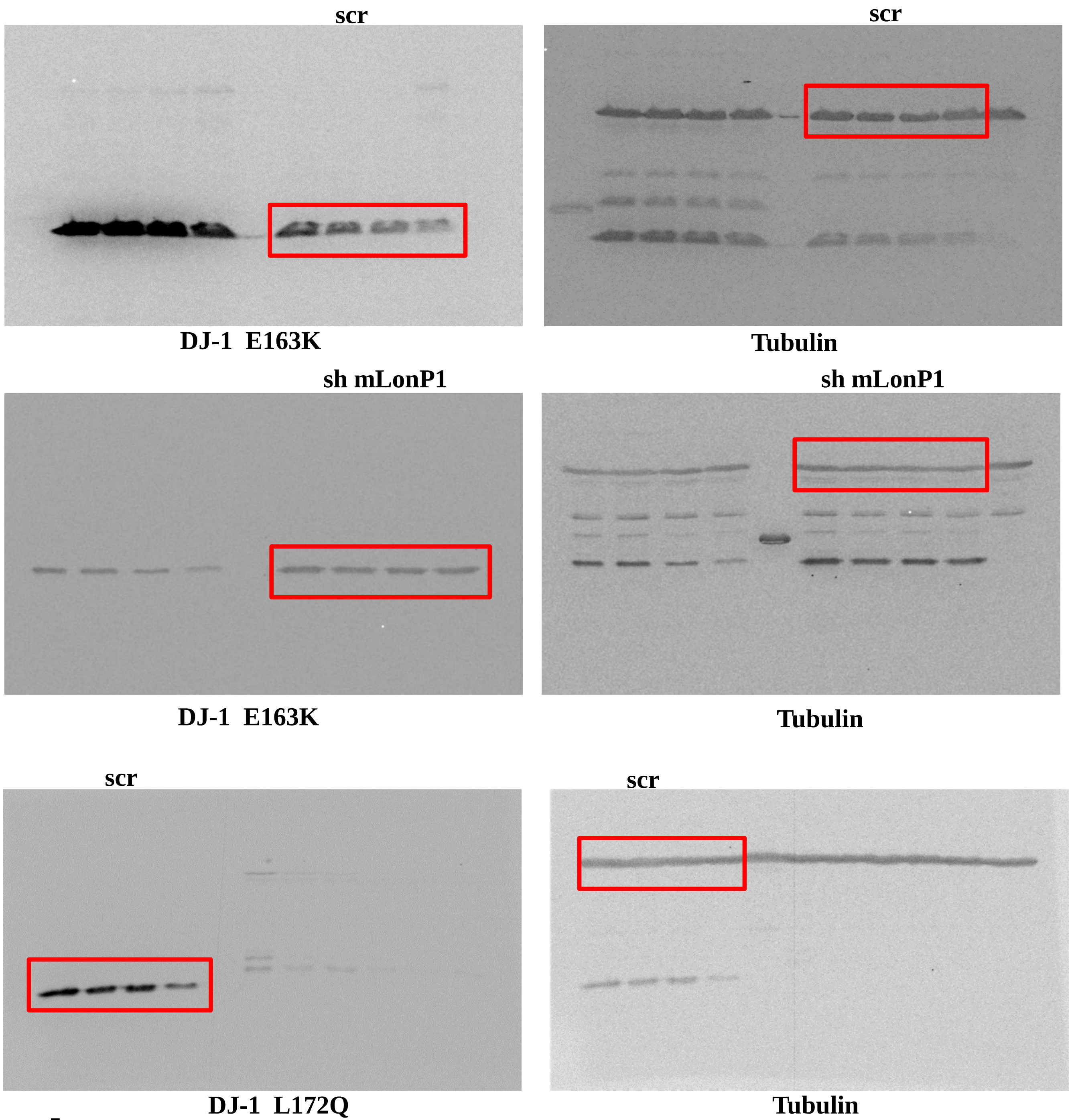

Supplementary Figure 5

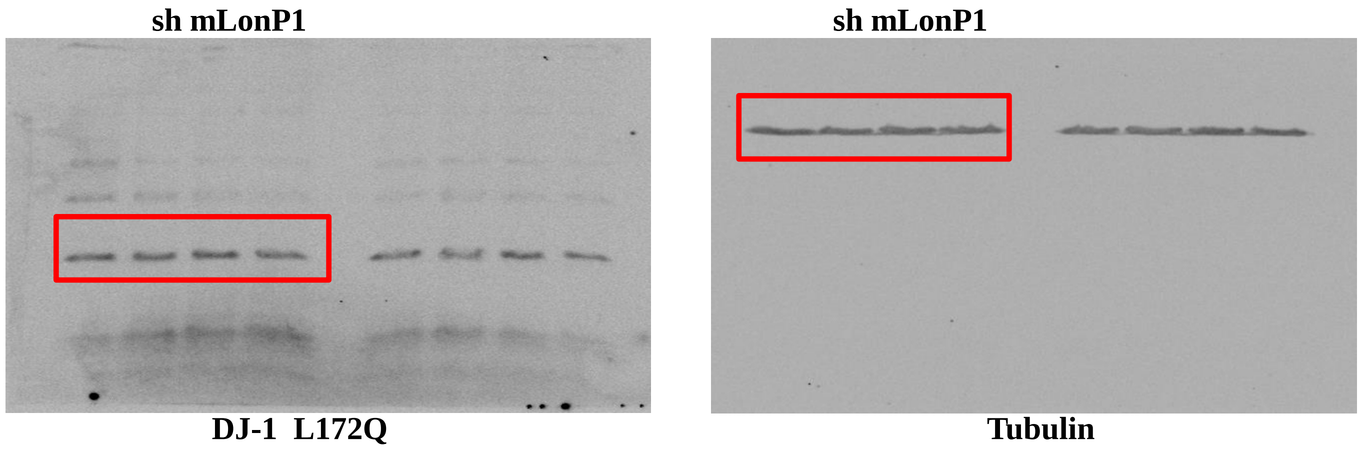

Supplementary Figure 5
